# Y_2_O_3_–Al_2_O_3_ microsphere crystallization analyzed by electron backscatter diffraction (EBSD)

**DOI:** 10.1038/s41598-020-67816-7

**Published:** 2020-07-06

**Authors:** Wolfgang Wisniewski, Peter Švančárek, Anna Prnová, Milan Parchovianský, Dušan Galusek

**Affiliations:** 1 Centre for Functional and Surface Functionalized Glass, Alexander Dubček University of Trenčín, 911 50 Trenčín, Slovakia; 2 Joint Glass Centre of the IIC SAS, TnU AD, and FChFT STU, Študentská 2, 911 50 Trenčín, Slovakia

**Keywords:** Glasses, Ceramics

## Abstract

The crystallization of glass microspheres in the Y_2_O_3_–Al_2_O_3_-system produced from precursor powders of four different nominal compositions via flame synthesis is analyzed in detail by electron microscopy with a focus on electron backscatter diffraction (EBSD). Growth models are formulated for individual microspheres crystallized during flame synthesis as well as after an additional heat treatment step. 16 different types of crystallized bodies are cataloged for future reference. They are presented without regard for their relative occurrence; some are extremely rare but illustrate the possibilities of flame synthesis in the analyzed system. All three phases in the binary Y_2_O_3_–Al_2_O_3_-phase diagram (Y_3_Al_5_O_12_, YAlO_3_ and Y_4_Al_2_O_9_) and α-alumina are located by EBSD. Energy dispersive X-ray spectrometry results obtained from these microspheres show that their chemical composition can deviate from the nominal composition of the precursor powder. The multitude of differing microsphere types showing polygon and dendritic crystal growth as well as phase separation indicate that flame synthesis can lead to a wide variety of parameters during microsphere production, e.g. via irregular flight paths through the flame, contaminants or irregular cooling rates.

## Introduction

Highly crystalline materials in the Y_2_O_3_–Al_2_O_3_ system have been intensely studied because cubic Y_3_Al_5_O_12_ (YAG) and orthorhombic YAlO_3_ (YAP) have been of interest as e.g. solid state laser materials for many years. The third chemical composition described in the system is monoclinic Y_4_Al_2_O_9_ (YAM) which shows a phase transition to a low-temperature modification of the same space group at 1,370 °C^1^. A further phase has been indicated^2^ but remains undescribed to the best of our knowledge. Melts of the Y_2_O_3_–Al_2_O_3_ system show polymorphism^3–5^ including the probable formation of two immiscible liquids after the melting of YAG which crystallized to form YAP and Al_2_O_3_^4,5^. Applying very large undercooling temperatures has also been described to cause a density driven phase separation^5^.

While single crystals and transparent ceramics are usually used as the host matrix for the optically active dopants allowing various applications, these materials are costly and time consuming to fabricate. An alternative approach of producing YAG is the controlled crystallization of glasses where the high melting temperature (T_m_) of YAG (calculated to be 2,213 °C^6^) combined with its high tendency towards spontaneous crystallization during cooling make glass production difficult. While YAG has been crystallized from multiple glasses, their crystallization e.g. in glasses also containing SiO_2_ often does not lead to the sole formation of YAG^7–9^ or shows a low nucleation rate^10^. Both effects are problematic with respect to the transparency of the resulting materials. Applying levitation melting and reducing the SiO_2_-content to only 4 wt% enables to produce glasses free of crystals^11^. The nano-crystallization of a glass containing an excess amount of Al_2_O_3_ to allow glass formation has recently allowed the fabrication of transparent YAG-Al_2_O_3_-nano ceramics without the need of elevated pressures^12^.

Utilizing the method of flame synthesis to produce glass microspheres provides the high cooling rates necessary for preventing crystallization during glass production^13–25^ while simultaneously limiting the maximum crystallite size to the maximum diameter of the respective sphere. Precursor powders in the wt% composition range (25–57) Y_2_O_3_–(43–75) Al_2_O_3_ have been processed using flame synthesis^13–21^, CeO_2_ has also been added to the composition^17^. Only the eutectic composition 40 Y_2_O_3_–60 Al_2_O_3_ and the composition doped with CeO_2_ led to XRD-amorphous microspheres, glass transition temperatures (T_g_) of 870–898 °C and primary crystallization temperatures (T_x_) of 899–936 °C could be measured from the produced spheres^13–21^. Secondary crystallization events between 996 and 1,020 °C were also observed in some glasses^20,21^. Powders of various nominal compositions in the Y_2_O_3_–Al_2_O_3_-system have been prepared using a combination of the Pechini method^26^ and flame synthesis to enhance the chemical homogeneity^18–21^.

The produced microspheres range from 10 to 40 µm in diameter^13–17,19^. X-ray diffraction (XRD) analyses of the microspheres mainly indicated the presence of YAG and trigonal α-Al_2_O_3_ which was only identified after subsequent thermal treatments at 1,300 °C or higher^13–15,17–20^. If any modifications of Al_2_O_3_ occurred at lower temperatures, their amounts were below the detection threshold of the performed XRD-experiments. While traces of YAP^13^ and δ-Al_2_O_3_^13,14^ have been reported, YAM has not been confirmed so far. Traces of Θ-Al_2_O_3_ were detected during high temperature (HT) XRD-measurements^18^. An infrared-spectrum of microspheres prepared using the stoichiometric composition has been presented^15^. Photo luminescence spectra of the Ce^3+^ doped composition have been presented^17^.

In a second process step, a bulk material may be prepared by pressure assisted sintering of the spheres via viscous flow in the temperature interval between T_g_ and T_x_. Sintering temperatures have ranged from 840 to 1,600 °C^13,15,16,21^ while pressures of 30–80 MPa^13,15,16,21^ have been applied. Hot pressing at 1,600 °C was reported to yield fully dense ceramics containing YAG and Al_2_O_3_ with a hardness of up to 18.0 ± 0.7 GPa^16,21^.

The microstructure inside crystallized microspheres produced using glasses in the Y_2_O_3_–Al_2_O_3_-system, however, has barely been analyzed. So far, microsphere cross sections have only been analyzed via SEM-micrographs^18–21^ with their limited information concerning phase attribution. Complementary energy dispersive X-ray spectrometry (EDXS) analyses^18^ are of limited value here as the microstructure components observed in the spheres are often smaller than the information volume of EDXS which extends beyond 1 µm for most measurements. By contrast, electron backscatter diffraction (EBSD) enables to locally analyze not only crystal orientations and orientation relationships amongst grains, but also phase distributions in the sub-µm scale using the crystallographic information acquired from the crystal lattices in the microstructure. The information depth of EBSD has been determined to be smaller than 100 nm in Si when using acceleration voltages of 20 kV or less and depends on the acquired EBSD-pattern quality^27^. The data evaluated from high-quality patterns originates from a smaller volume than that of low quality patterns^27^. EBSD has only been applied to a microsphere in the Y_2_O_3_–Al_2_O_3_-system once ^21^ and the details of this analysis will be presented below.

The recent application of EBSD to crystallized glasses has led to many new insights in the respectively analyzed materials systems. For example, fresnoite containing glass–ceramics have been intensely studied using EBSD^28–31^ and the results were summarized in a recent review^28^. EBSD has also been used to analyze YAG-containing glass–ceramics^7,8,10^, crystal growth mechanisms^28^, crystal growth in phase separating glasses^30,31^ and much more.

This current article presents scanning electron microscopy (SEM), EDXS, and especially EBSD results illustrating the microstructure in a wide variety of microsphere types resulting from the flame synthesis of precursor powders with four different nominal compositions in the Y_2_O_3_–Al_2_O_3_-system.

## Experimental procedure

Precursor powders with ma% compositions of 40–69 Y_2_O_3_ and 31–60 Al_2_O_3_ were prepared using a modified Pechini sol–gel method^26^. Aluminium nitrate (Al(NO_3_)_3_, 99.9%, Sigma Aldrich, Germany) was dissolved in deionized water and mixed with an yttrium nitrate solution prepared by the dissolution of Y_2_O_3_ (99.9%, Treibacher Industry, Austria) in diluted HNO_3_. Subsequently, an aqueous solution of citric acid and ethylene glycol in the molar ratio 1:1 was added. The mixtures were refluxed at 85 °C for 2 h and then heated to 150 °C to promote polymerization and evaporate the solvent. The viscosities of the solutions increased rapidly to form aerated resins from which the organic compounds were removed via calcination at 800 °C for 6 h in an ambient atmosphere.

The precursor powders for flame synthesis were obtained by sieving through a 40 μm sieve and then feeding them into a methane-oxygen flame where it is assumed that they melted and formed droplets subsequently quenched using deionized water to produce a batch of microspheres. The collected batches were dried and calcined at 650 °C for 4 h to remove any organic residue. Selected, and subsequently noted, microsphere batches were crystallized for up to 6 h at up to 1,600 °C in an electrical Classic CZ tube furnace using a heating rate of 20 °C/min. After these treatments, the furnace was switched off, allowing a slow cooling rate of 40 °C/min.

In order to perform analyses in the SEM, the microspheres were embedded using a Buehler KonductoMet (heated to 150 °C for 1 min) and a cross section was prepared by grinding with decreasing grain sizes down to 0.5 µm using a Buehler AutoMet 300 polisher. A final finish of up to 6 h vibration polishing using a Buehler VibroMet 2 (200 g static load) and a MasterPrep polishing suspension (50 nm sol–gel alumina) was applied. Polished cross sections were coated with a thin layer of Au at about 5 Pa to avoid surface charging.

SEM analyses were performed using a Jeol JSM 7600F SEM equipped with an X-Max 50 mm^2^ EDXS detector and a Nordlis Max EBSD-camera, both from Oxford Instruments. EDXS-spot measurements were acquired using an acceleration voltage of 10 kV. EBSD-scans were performed using an acceleration voltage of 15 kV and a current of up to ca. 3 nA. The scans were captured using the OxfordInstruments software Aztec 3.1 and evaluated using the Channel 5 software package. Pseudosymmetries in datasets containing data points indexed as orthorhombic YAP were cleaned so as to disregard 90° rotations around the 10–1, -101, -10–1, 030 and 0–30 axes allowing a deviation of 5°.

## Results and discussion

Crystallized microspheres have been produced using glasses of various compositions in the Y_2_O_3_–Al_2_O_3_-system^13–16,18–21^. They have been shown to contain YAG and α-Al_2_O_3_^13–15,18–21^ while traces of YAP have only been reported once^13^. Although various microstructures in cross sections of microspheres have been presented using SEM^18–21^, they have never been analyzed in detail using EBSD which was only recently applied to a microsphere containing YAG^21^.

**Figure 1:**
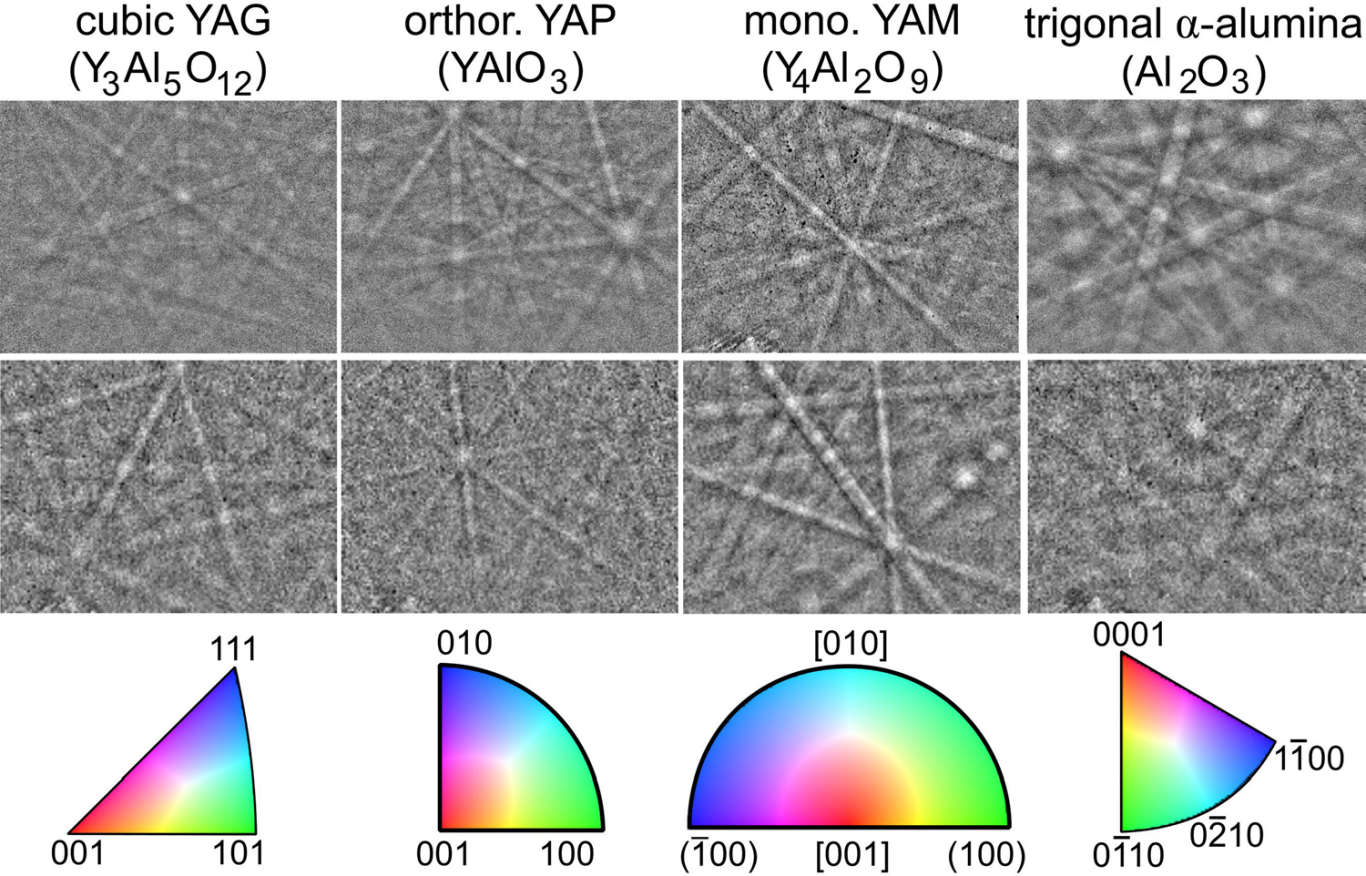
EBSD-patterns of the respectively stated phases acquired from polished cross sections of microspheres in the Y_2_O_3_–Al_2_O_3_-system with a binning of 1 × 1 (top) and the binning of 4 × 4 (bottom). IPF-legends of the respective symmetries are presented below for future reference.

Figure 1 presents EBSD-patterns acquired from the analyzed microspheres and respectively attributed to YAG (indexed using ICSD file no. 67102), YAP (indexed using ICSD file no. 4115), YAM (indexed using ICSD file no. 51077) or α-alumina (indexed using the corresponding material file from the hkl-database). The patterns in Fig. 1 represent the pattern quality obtainable when using the minimum binning of 1 × 1 or the binning of 4 × 4 used when acquiring EBSD-scans from the subsequently analyzed microspheres after the applied polishing procedure. Inverse Pole Figure (IPF) legends or the respective symmetries are presented below for subsequent reference. While YAG has been extensively analyzed using EBSD including in glass-ceramics^7–10^ and an EBSD-pattern of YAP has been presented^32^, we failed to find any literature reporting EBSD-results of YAM.

**Figure 2:**
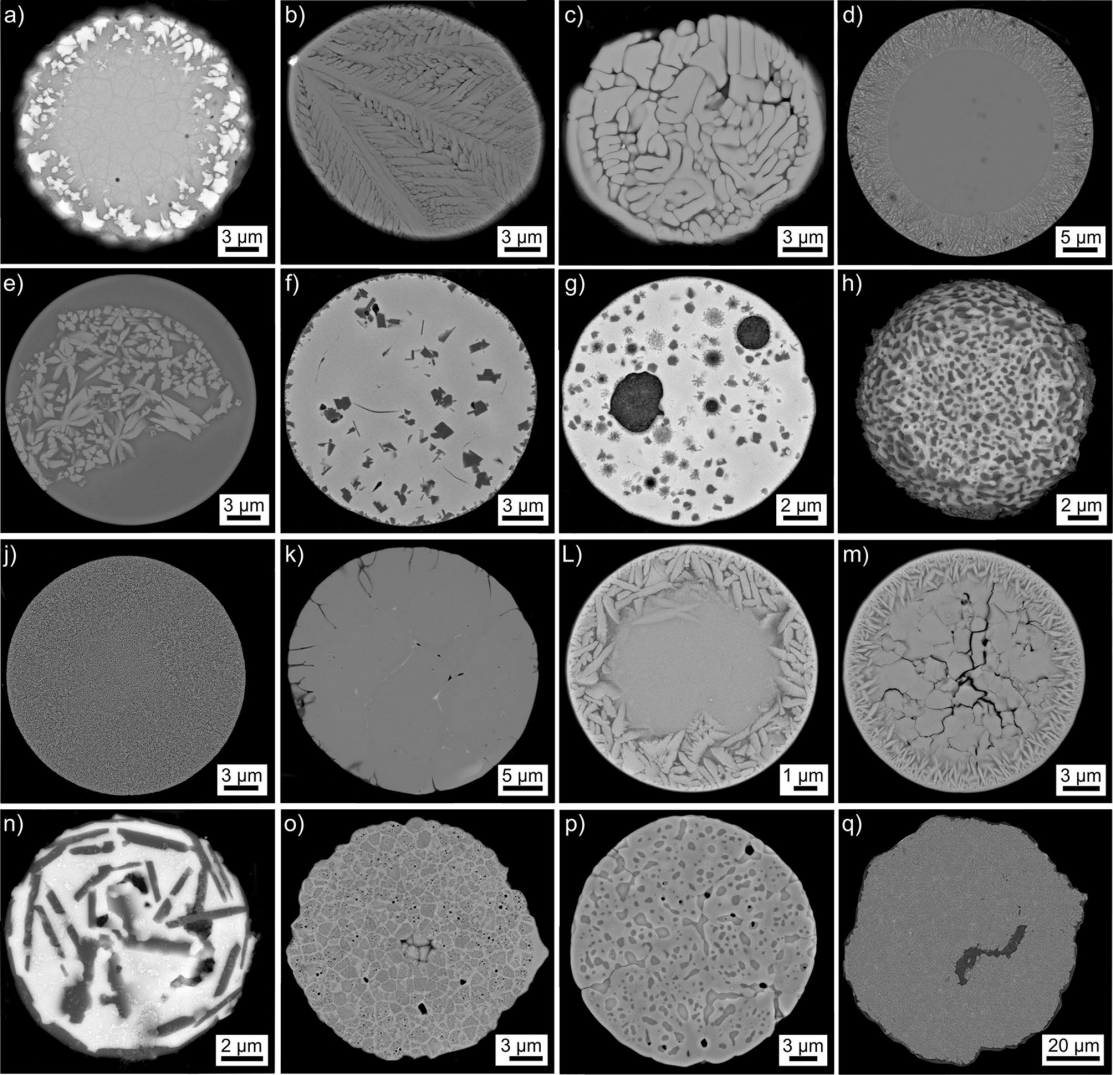
SEM-micrographs (**a**)–(**q**) presenting cross sections through microspheres produced via the flame synthesis of precursor powders in the Y_2_O_3_–Al_2_O_3_-system.The nominal chemical compositions and thermal treatments during production are stated in Table 1. Chemical compositions measured by EDXS in selected microspheres are presented in Table 2.

An overview featuring SEM-micrographs of the microsphere morphologies subsequently analyzed in detail in this manuscript is presented in Fig. 2. The parameters applied during the production of the analyzed microsphere batches are summarized in Table 1, the respective contents of Y, Al and O in at% are presented for an easy comparison with the subsequent EDXS-results. A rough estimate of the respective occurrence of the presented microstructures in the analyzed batches is also stated, but it should be noted that varying parameters may significantly change these occurrences. Singularities refer to microstructures only observed once during the performed analyses. Rarities were observed more than once but are estimated to occur in less than 1% of the analyzed microspheres of the batch. Dominant microstructures represent the majority of the microspheres in an annealed batch while secondary microstructures frequently occur, but less often than the dominant microstructure in the annealed batch.

**Table 1:** Nominal precursor powder compositions in ma%, the respective elements in at%, crystallization temperatures T_cryst._ and crystallization times t_cryst._ used to produce the microspheres presented in Fig. 2.

Figure	Y_2_O_3_ (ma%)	Al_2_O_3_ (ma%)	Y (at%)	Al (at%)	O (at%)	T_cryst._ (°C)	t_cryst._ (h)	Rel. occurrence
2a	40	60	9.3	30.7	60.0	–	–	Rarity
2b	57	43	15.0	25.0	60.0	–	–	Rarity
2c	57	43	15.0	25.0	60.0	–	–	Rarity
2d	57	43	15.0	25.0	60.0	–	–	Rarity
2e	57	43	15.0	25.0	60.0	–	–	Singularity
2f	40	60	9.3	30.7	60.0	1,000	4	Dominant
2g	40	60	9.3	30.7	60.0	1,000	4	Dominant
2h	40	60	9.3	30.7	60.0	1,000	4	Rarity
2j	40	60	9.3	30.7	60.0	1,300	0.5	Rarity
2k	40	60	9.3	30.7	60.0	1,500	6	Dominant
2L	69	31	20.1	19.9	60.0	1,600	4	Rarity
2m	57	43	15.0	25.0	60.0	915	0.33	Rarity
2n	57	43	15.0	25.0	60.0	915	0.33	Singularity
2o	69	31	20.1	19.9	60.0	1,600	4	Secondary
2p	69	31	20.1	19.9	60.0	1,600	4	Dominant
2q	40	60	9.3	30.7	600	1,500	6	Rarity

Chemical compositions measured from areas in selected microspheres via EDXS are summarized in Table 2, showing that significant deviations from the nominal powder compositions occur.

**Table 2:** Chemical compositions determined from selected microspheres via EDXS, the ideal compositions of YAG, YAP and YAM are stated for comparison.

Figure	Y (at%)	Al (at%)	O (at%)	Impurities
2a (bright)	16.7	26.6	56.7	
2a (dendritic)	10.4	32.4	57.2	
2c	22.4	22.2	55.4	
2d (cryst.)	6.5	35.9	57.6	
2d (uncryst.)	6.0	37.0	57.0	
2e (cryst.)	29.5	17.7	52.4	0.4 Si
2e (glass)	19.1	27.3	53.4	0.2 Si
2f (bright)	11.6	31.6	56.8	
2f (dark)	2.2	38.6	59.2	
2h	9.4	34.7	55.9	
2j	10.4	32.5	57.1	
2k	18.1	26.9	55.0	
2L (bright)	24.9	20.0	55.1	
2L (dark sphere)	10.8	32.9	56.3	
2m	19.7	27.2	53.1	Fe
2n (bright)	13.0	31.4	54.7	0.2 Mg, 0.7 Ca
2n (dark)	5.4	35.4	56.9	0.6 Mg, 1.7 Ca
2o	15.6	28.7	55.7	Fe
2p (YAP)	27.6	17.3	55.1	Fe
2p (sec. phase)	25.5	19.3	55.2	Fe
Ideal composition:				
YAG (Y_3_Al_5_O_12_)	15.00	25.00	60.00	
YAP (YAlO_3_)	20.00	20.00	60.00	
YAM (Y_4_Al_2_O_9)_	26.67	13.33	60.00	

### Crystallization observed directly after production (no heat treatment)

**Figure 3:**
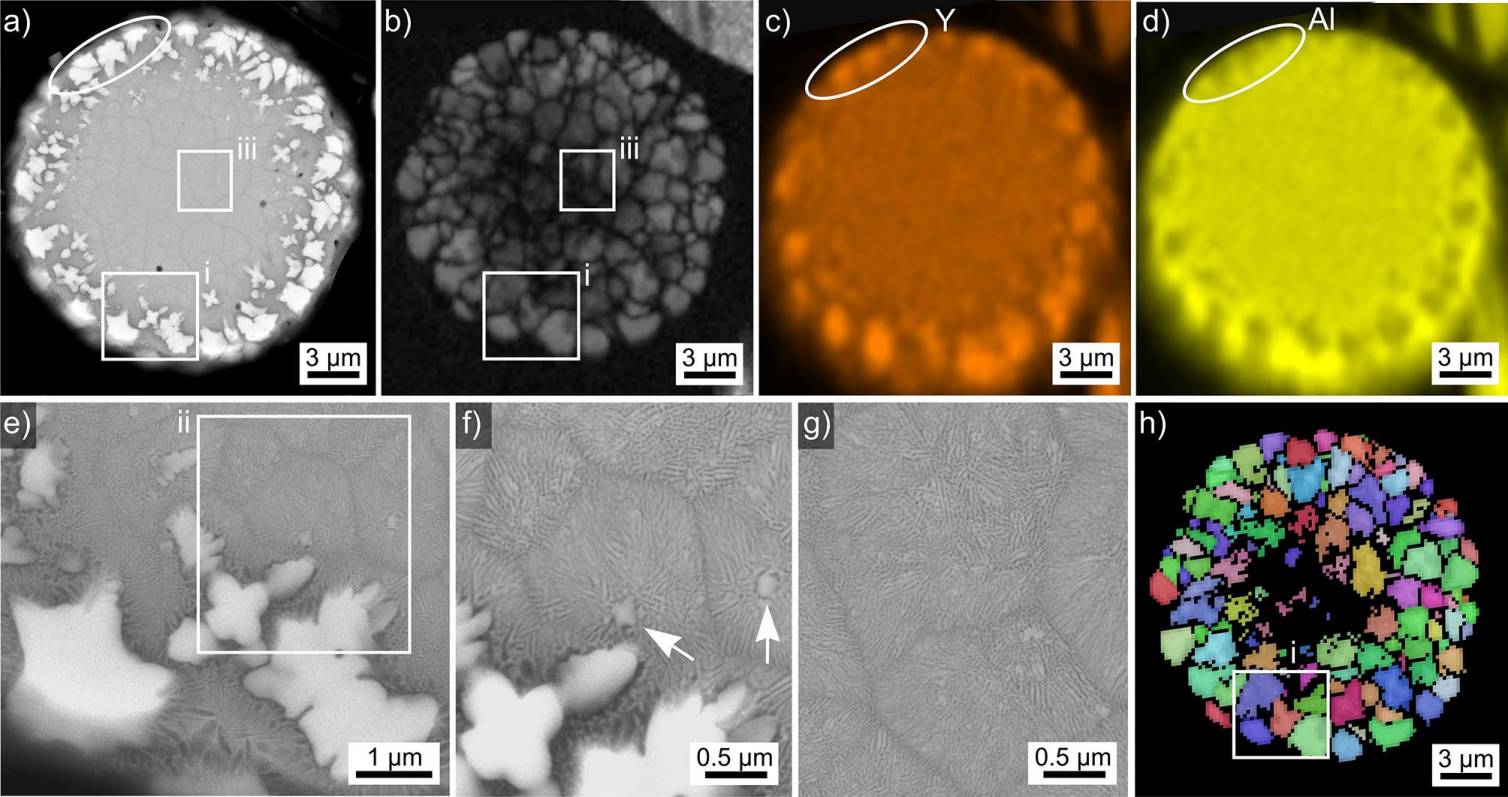
Results obtained from the microsphere type introduced in Fig. 2a: (**a**) SEM-micrograph, (**b**) BC-map of a performed EBSD-scan and the element maps (**c**) of Y and (**d**) of Al acquired using EDXS during the scan. A selected area is circled for comparison. (**e**) The area in frame (i) presented in greater detail. (**f**) the area in frame (ii) and (**g**) the area in frame (iii) presented with the same magnification, the arrows highlight compact segments. (**h**) BC + IPF-map of the performed EBSD-scan after only applying the wild spike cleanup, the IPF-legend for cubic phases like YAG is presented in Fig. 1.

Results obtained from the microsphere type introduced in Fig. 2a are presented in Fig. 3. It is the same microsphere also featured in Ref. ^21^ which illustrated that more than 100 amorphous spheres of the batch were scanned in order to locate this one. The SEM-micrograph in Fig. 3a shows bright structures near the periphery but a barely discernible grain structure in the bulk. The band contrast (BC)-map of an EBSD-scan performed in this sphere in Fig. 3b confirms that it is fully crystallized and the grain structure appears much more homogeneous than the contrast in the SEM-micrograph of Fig. 3a, all indexed data points are attributed to YAG. The element maps of Y in Fig. 3c and Al in Fig. 3d illustrate that Y is enriched where Al is depleted. Comparing the circled area in various figures confirms that the large, bright YAG crystals in the SEM-micrograph provide an elevated Y signal while Al is depleted in comparison to the YAG grains in the bulk. Furthermore, Al shows the strongest signal in the vicinity of the bright YAG crystals.

The area in frame (i) is presented in greater detail in Fig. 3e where dark areas are clearly discernible around the bright crystals. Furthermore, the outmost bright crystals show a large, homogeneous core while the smaller bright crystals further in the bulk show morphologies indicating dendritic growth. The area in frame (ii) is presented in Fig. 3f while Fig. 3g presents the area in frame (iii) with the same magnification. Both figures show that the “grains” in the bulk are in fact composed of fine, often parallel structures in the nm-scale similar to the lamellae observed after eutectic crystallization^33^. Furthermore, the lamellae often seem to originate from a more homogeneous core, e.g. highlighted by the white arrows. Furthermore, thin lamellae penetrate the dark areas around the bright crystals, very similar to the honey comb structures observed in fresnoite glass ceramics grown via electrochemically induced nucleation (EiN)^29^.

Figure 3h features a combined BC + IPF-map of the performed EBSD-scan. It shows that the structures in the bulk show only a single crystal orientation, meaning the crystal lattices are connected and probably grew via the dendritic mechanism. Furthermore, the orientation domains e.g. observed in frame (i) are clearly larger than the bright crystals in the same area framed in Fig. 3a. Hence the homogeneous crystals are in an epitaxial relationship with the fine structures surrounding them in analogy to the fresnoite glass–ceramics featured in Ref. ^29^.

The chemical composition of the homogeneous YAG-grains in this microsphere averaged over 5 EDXS-spot measurements was measured to be 16.7 Y–26.6 Al–56.7 O at%, which is close to the theoretical composition of YAG. Comparable measurements obtained from the lamellar structures in the bulk provide a composition of 10.4 Y–32.4 Al–57.2 O at%, i.e. a significantly elevated level of Al. Considering the information volume of EDXS, it may be concluded that the dark phase in the dendritic orientation domains is enriched in Al_2_O_3_ while the bright lamellae are composed of YAG and allow the acquisition of EBSD patterns.

The results presented above show that the melt forming this sphere crystallized at a very high temperature while cooling from the outside, which is in agreement with the lack of any heat treatment after production. The homogeneous, polygon YAG crystals in the periphery formed when their matrix was relatively hot and crystal growth was slow, also allowing the expulsion of access Al_2_O_3_. The later formed a diffusion barrier, limiting further growth. As the undercooling rate increased during cooling, thin channels of YAG managed to bridge this barrier while the temperature further in the bulk was still too high to allow nucleation. When these channels reached the uncrystallized matrix not enriched in Al_2_O_3_, the undercooling rate was large enough for faster crystal growth, causing the mechanism change to dendritic growth. Instead of accumulating undesirable elements in a growth front, dendrites can simply grow around them, forming pockets of residual material. In the current case, the latter is enriched in Al_2_O_3_, but whether it is crystalline or not is impossible based on the presented data.

When the temperature finally dropped low enough for nucleation in the bulk, the remaining melt in the sphere crystallized, starting from locations like the compact cores highlighted by the white arrows in Fig. 3. Directly comparing Fig. 3f to Fig. 3g allows the impression that the orientation domains in Fig. 3g, i.e. near the center of the sphere, are less ordered. This would explain the lower EBSD-pattern quality and indexing problems indicated to occur here by the Fig. 3b and Fig. 3h. Similarly fine structures have failed to provide any EBSD-patterns in fresnoite glass–ceramics showing phase separation in addition to fine dendritic growth^31^.

**Figure 4:**
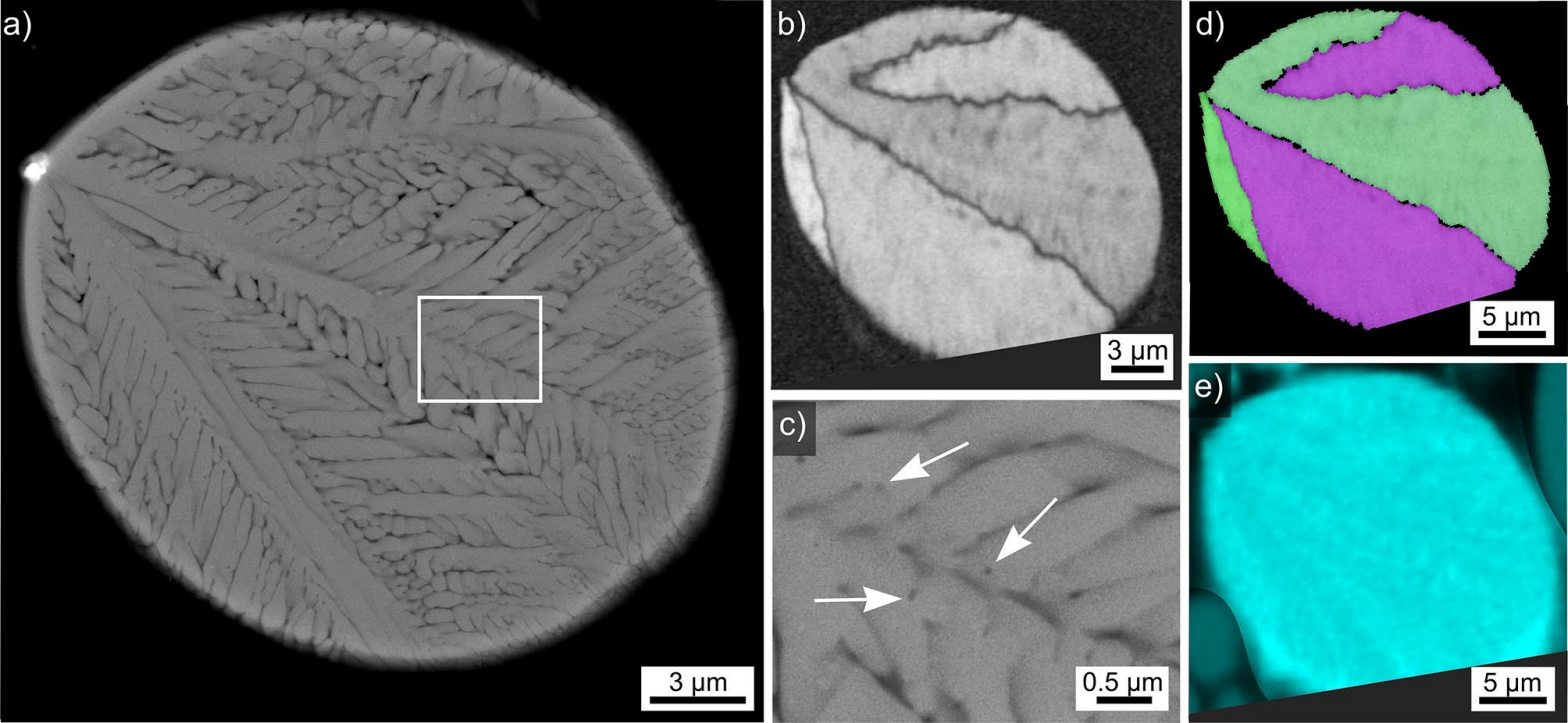
Results obtained from the microsphere type introduced in Fig. 2b: (**a**) SEM-micrograph and (**b**) BC-map of an EBSD-scan performed on the area. (**c**) SEM-micrograph of the framed area in greater detail. (**d**) IPF + BC-map of the EBSD-scan and (**e**) element map of Al. The IPF-legend for cubic phases like YAG is presented in Fig. 1.

Results obtained from the microsphere type introduced in Fig. 2b are presented in Fig. 4. The SEM-micrograph in Fig. 4a features a cross section clearly indicating dendritic growth with a relatively high velocity. All indexed EBSD-patterns acquired from this microsphere were attributed to YAG. The BC-map of an EBSD-scan performed on the area is presented in Fig. 4b and supports this impression as only four domains of homogeneous band contrast are indicated. However, the interdendritic areas are too small to be clearly resolved by the scan performed using a step size of 200 nm. Decreasing the step size led to drift during further scan attempts. The framed area in Fig. 4a is presented in greater detail in Fig. 4c to show that the YAG crystals contain nm-scale inclusions e.g. highlighted by the white arrows. The IPF + BC-map of the performed EBSD-scan presented in Fig. 4d confirms the dendritic growth as only three orientation domains occur in the analyzed cross sections. A similar microsphere showing a single crystal orientation in the prepared cross section has been measured, but Fig. 4 illustrates that this is insufficient data for claiming it to be a “single crystal microsphere”.

The implied low nucleation rate but high crystal growth velocity allow the conclusion that this sphere also crystallized at a high temperature, but with a higher undercooling rate then the sphere featured in Fig. 3. While e.g. only a few, very large dendrites of YAG have been observed in a SiO_2_/Al_2_O_3_/Y_2_O_3_/CaO glass after controlled crystallization^10^, the lack of any heat treatment after the flame synthesis enables to conclude that this sphere also crystallized before it was quenched in water. The element map of Al presented in Fig. 4e shows very little contrast but it is discernible that the amount of Al is lower in the YAG-dendrites than in the interdendritic spaces, indicating Al was again accumulated outside of the crystal lattice.

**Figure 5:**
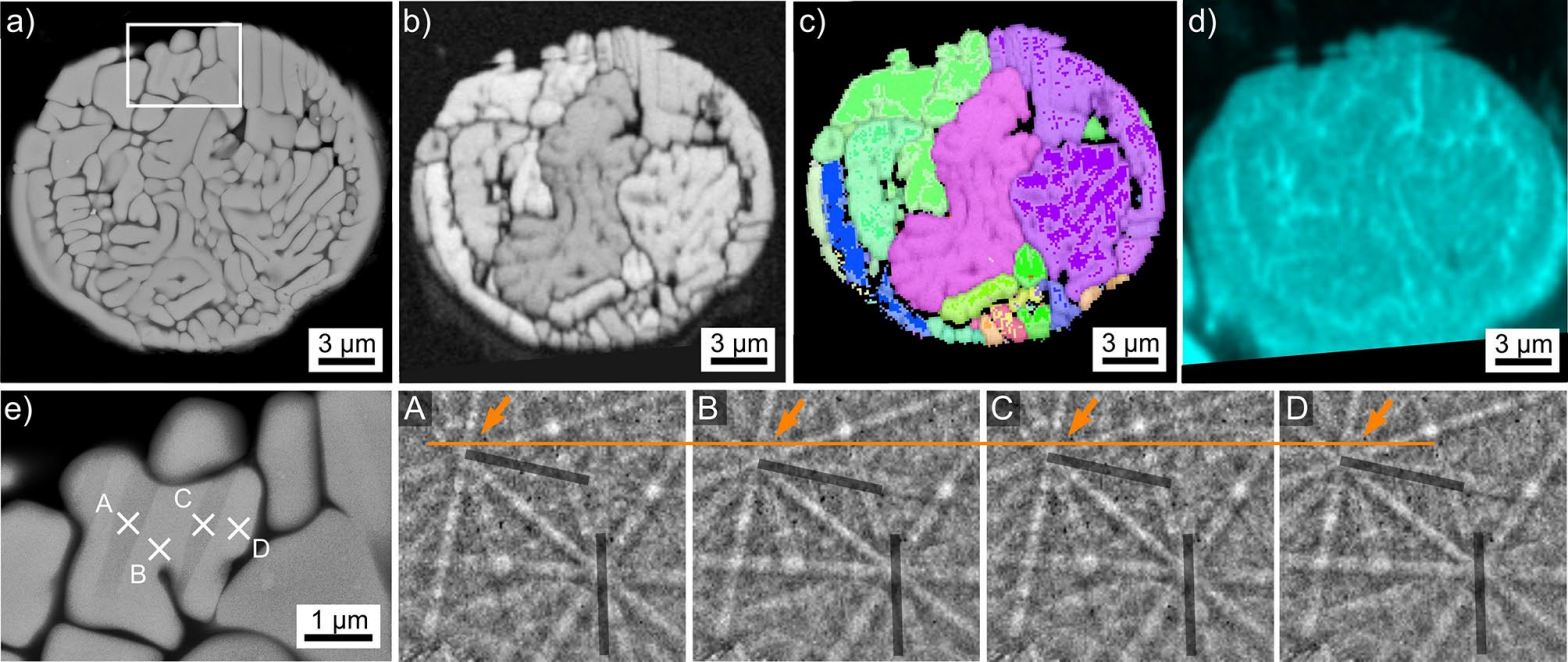
Results obtained from the microsphere type introduced in Fig. 2c: (**a**) SEM-micrograph. (**b**) BC-map of an EBSD-scan performed on the area. (**c**) IPF + BC-map of the same scan, the IPF-legend for orthorhombic phases like YAP is presented in Fig. 1. (**d**) Element map of Al acquired by EDXS during the EBSD-scan. (**e**) Framed area in greater detail, the EBSD-patterns 5A–5D originate from the locations A–D. The orange line serves as reference line for the eye while the dark lines highlight selected Kikuchi bands.

The SEM-micrograph of the microsphere introduced in Fig. 2c and also presented in Fig. 5a shows a homogeneous SEM-contrast of the growth structures and again implies that some of them may be connected. All high-quality EBSD-patterns acquired from this sphere were indexed as YAP. The BC-map of an EBSD-scan performed on this sphere is presented in Fig. 5b. It shows larger areas of more diverse contrast, supporting the concept of links between the orientation domains. The contrast between them would then be caused by slight differences in the EBSD-patterns acquired from these regions. The IPF + BC-map of the performed EBSD-scan confirms the orientational link of many “grains” in the SEM-micrograph, proving that these structures are YAP dendrites. YAP dendrites have also been described after the crystallization of YAG single crystals where alumina was additionally detected^4^. The absence of alumina indicates that these YAP crystals grew in the melt without first forming YAG. The EDXS-map in Fig. 5d was acquired during the EBSD-scan and shows that Al is again enriched in the interdendritic spaces. As the latter also fail to provide EBSD-patterns in the BC-map, it is likely that they contain residual glass.

Figure 5e presents the region framed in Fig. 5a in greater detail to illustrate broad, parallel bands of darker SEM-contrast in the highlighted grain which appears homogeneous in the IPF + BC-map of Fig. 5c after the applied cleaning procedure. The EBSD-patterns 5A-5D were acquired at the locations A-D: pattern 5A and 5C originate from the dark bands while the patterns 5B and 5D represent the slightly brighter bulk of the grain. The orange line is superimposed onto the EBSD-patterns to illustrate that the position of the zone axis highlighted by the arrow shifts: it is slightly further from the line in the patterns 5B and 5D than in the patterns 5A and 5C. Furthermore, two Kikuchi-bands have been highlighted by dark lines to illustrate that these bands vary significantly in intensity. The close to vertical band is stronger in the patterns 5A and 5C while the close to horizontal band is stronger in the patterns 5B and 5D. The orientation relationship between these orientation domains is a 90° rotation around the 0$\bar{3}$0-axis, explaining why it appears homogeneous in the cleaned IPF + BC-map: this orientation is considered in the applied pseudo-symmetry cleanup. However, given the parallel bands in the SEM-micrograph of Fig. 5e and the outlined details in the EBSD-patterns 5A-5D, it is plausible to conclude that the featured grain contains a crystallographic {030}-twin of YAP. This would be rather interesting as {101} twins have been described to dominantly occur e.g. in YAP single crystals^34^.

The chemical composition of the YAP-grains in this microsphere averaged over 5 EDXS-spot measurements is 22.4 Y–22.2 Al–55.4 O at%, which is close to the theoretical composition of YAP. The interdendritic areas are too small for reliable EDXS-measurements in the SEM.

The coarse-structured dendrites in the presented microsphere indicate a low nucleation rate but high crystal growth velocity. As this sphere was also not heat treated after flame synthesis, it also grew at high temperatures similar to the sphere featured in Fig. 4 or the fresnoite dendrites grown via EiN^28,29^.

**Figure 6:**
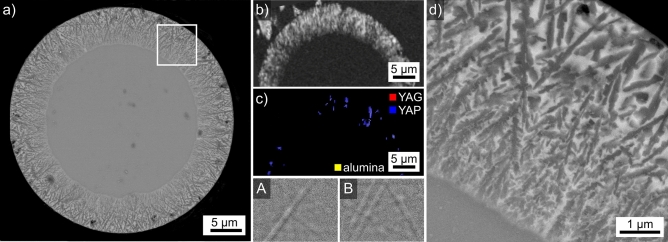
Results obtained from the microsphere type introduced in Fig. 2d: (**a**) SEM-micrograph overview. (**b**) Part of the BC-map of an EBSD-performed in the area. (**c**) Phase-map of the area featured in (**b**). (**d**) SEM-micrograph of the framed area in greater detail. The EBSD-patterns 6A and 6B were obtained from the outer, crystallized area of the sphere.

The SEM-micrograph of the microsphere introduced in Fig. 2d and also presented in Fig. 6a indicates a layer of surface crystallization growing into this sphere while significant bulk crystallization is not indicated in this cross section. Whether the dark areas in the bulk are crystals or a preparation artifact cannot be said at this point, they do not cause any significant signal during the subsequent EBSD and EDXS analyses. The BC-map of an EBSD-scan performed on the area and presented in Fig. 6b confirms that EBSD-patterns can be systematically acquired from this layer. The phase-map of the same area in Fig. 6c shows that only few data points are indexed and all of those are attributed to YAP. The EBSD-patterns 6A and 6B were acquired from the crystallized area and illustrate why indexing may be problematic: pattern 6A contains only very few Kikuchi-bands and pattern 6B indicates EBSD-pattern superposition.

Figure 6d presents the framed area in greater detail to show that the crystallized layer is actually composed of dark, dendritic growth structures while the interdendritic spaces show a brighter contrast in the SEM than the uncrystallized bulk. Furthermore, the dendrites become finer as the distance to the sphere surface increases, indicating a reducing growth velocity during crystallization. The chemical composition of the crystallized area in this microsphere averaged over 5 EDXS-spot measurements was measured to be 6.5 Y–35.9 Al–57.6 O at% while the uncrystallized area in the bulk shows the very similar composition 6.0 Y–37.0 Al–57.0 O at%.

Considering the high amount of Al in this microsphere and the relatively low backscatter signal, it is plausible to conclude that the dark structures are probably dendrites composed of an aluminum oxide. This is supported by their similarity to the structures which will be presented in Figs. 8 and 9 and also probably contain an aluminum oxide. Furthermore, the poor quality of the EBSD-patterns 6A and 6B is also similar to those featured in Fig. 8. As all indexable EBSD-patterns acquired from this sphere were attributed to YAP, the crystallization of an aluminum oxide from the glass shifts the composition of the remaining glass so that it crystallizes to form YAP.

**Figure 7:**
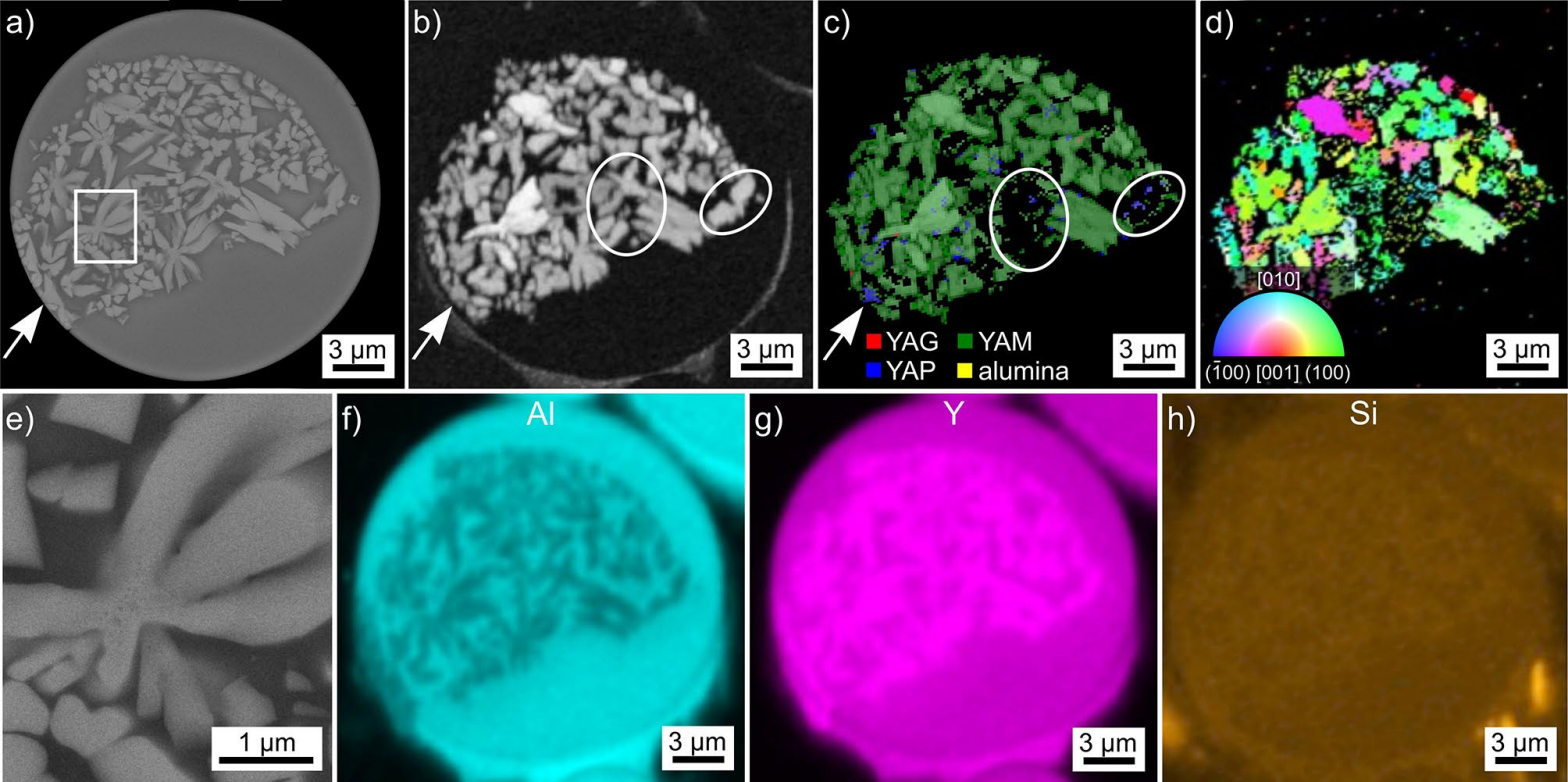
Results obtained from the microsphere type introduced in Fig. 2e: (**a**) SEM-micrograph overview. (**b**) BC-map, (**c**) phase + BC-map and (**d**) IPF + BC-map of an EBSD-scan performed on the area. The IPF-legend for monoclinic phases like YAM is presented in Fig. 1. (**e**) SEM-micrograph featuring the framed area in detail. The white arrows highlight a grain predominantly attributed to YAP while the circles highlight areas where the EBSD-patterns failed to be indexed. The element maps of (**f**) Al, (**g**) Y and (**h**) Si are presented.

The SEM-micrograph of the microsphere introduced in Fig. 2e is also presented in Fig. 7a where the inhomogeneous distribution of crystals indicates an inhomogeneous chemical composition in the sphere, e.g. a stria. These crystals provided high quality EBSD-patterns which, however, failed to be indexed as either YAG, YAP or α-alumina. Including the material file of monoclinic YAM led to indexed orientation solutions with mean angular deviation (MAD)-values below 1°. Examples of these patterns are presented in Fig. 1.

The BC-map of an EBSD-scan performed on the sphere is presented in Fig. 7b, confirming that crystals are only detected within the area indicating crystallization in the SEM-micrograph. The BC + phase map of this scan in Fig. 7c shows that most data points are attributed to YAM, but some are also attributed to YAP. The latter usually occur near the edges of YAM-grains, but the single grain highlighted by the white arrow in multiple figures is almost solely attributed to YAP. Furthermore, it is clear that some of the crystals providing a strong BC signal provided EBSD-patterns which were not indexed, two are circled in white. An IPF + BC-map of the EBSD-scan is presented in Fig. 7d to show that these crystals generally show independent orientations, i.e. grew via the polygon growth mechanism. Twinning as e.g. described for YAM grown via the Czochralski method^35^ is not indicated.

Attempting to obtain single EBSD-patterns from the grain indexed as YAP after performing two EBSD-scans was not possible because EBSD-patterns could no longer be acquired although the sample had remained in the SEM. Hence these phases are either sensitive to degradation by the electron beam or there was simply too much C accumulation during the performed scans. Alternatively, the energy induced by the electron beam could have enabled a phase transition from high-temperature YAM to low-temperature YAM^1^. As both apparently share the same space group^1^, EBSD cannot separate these modifications. The accompanying volume change of 0.4%^1^ would induce stresses to the crystal lattice, possibly preventing the formation of EBSD-patterns.

The area framed in Fig. 7a is presented in greater detail in Fig. 7e to show that these crystals also incorporated inclusions of darker contrast, i.e. perhaps pockets of residual glass. EDXS-data was acquired during the EBSD-scan. The element map of Al in Fig. 7f shows that these crystals show a lower amount of Al than their matrix. The latter shows no discernible difference to the Al-signal to the uncrystallized area in the sphere. The element map of Y presented in Fig. 7g shows the inverse result: Y is enriched in the crystals while the rest of the sphere again appears to have a homogeneous Y-content. The EDXS-signal of Si was very weak, but the detailed evaluation of acquired EDXS-spectra confirmed that minimal amounts of a Si-contamination occur in this sphere. The element map of Si in Fig. 7h shows only a slight contrast, but it is discernible that the distribution of Si matches that of Y, i.e. it is enriched in the crystals.

The composition of the crystals attributed to YAM in this microsphere averaged over 5 EDXS-spots was measured to be 29.5 Y–17.7 Al–0.4 Si–52.4 O at% which is near the theoretical at% composition of YAM stated in Table 2. The composition measured from the uncrystallized glass was 19.1 Y–27.3 Al–0.2 Si–53.4 O at%, i.e. 10 at% more Al but less Y and only ca. half the amount of Si detected in the crystals. Although the detected Si-impurity is minimal, e.g. adding only 4 wt% of SiO_2_ enabled the controlled production of a glass in this system using levitation melting^11^. Here Si was shown to be incorporated into the crystal structure of YAG^11^ during the glass crystallization. Figure 7h implies it may similarly be incorporated into YAM. As SiO_2_ can have an extreme effect on glass properties as e.g. the viscosity, the irregular area of crystallization in this sphere could be explained by an Si-enriched stria allowing crystallization during which Si is enriched in the crystals. Once the amount of Si in the residual matrix reaches that of the uncrystallized glass outside of the stria, the system “freezes” and crystallization is stopped.

While a Si depletion would decrease the viscosity in many glasses, considering the depletion of Si to enhance the viscosity in this sphere is plausible due to the high melting temperatures in the Y_2_O_3_–Al_2_O_3_ system. It is also plausible to assume the formation of local eutectic compositions in the ternary Y_2_O_3_–Al_2_O_3_-SiO_2_ system which should show lower viscosities at the same temperature than related compositions in the binary Y_2_O_3_–Al_2_O_3_ system.

### Crystallization observed after subsequent annealing

**Figure 8:**
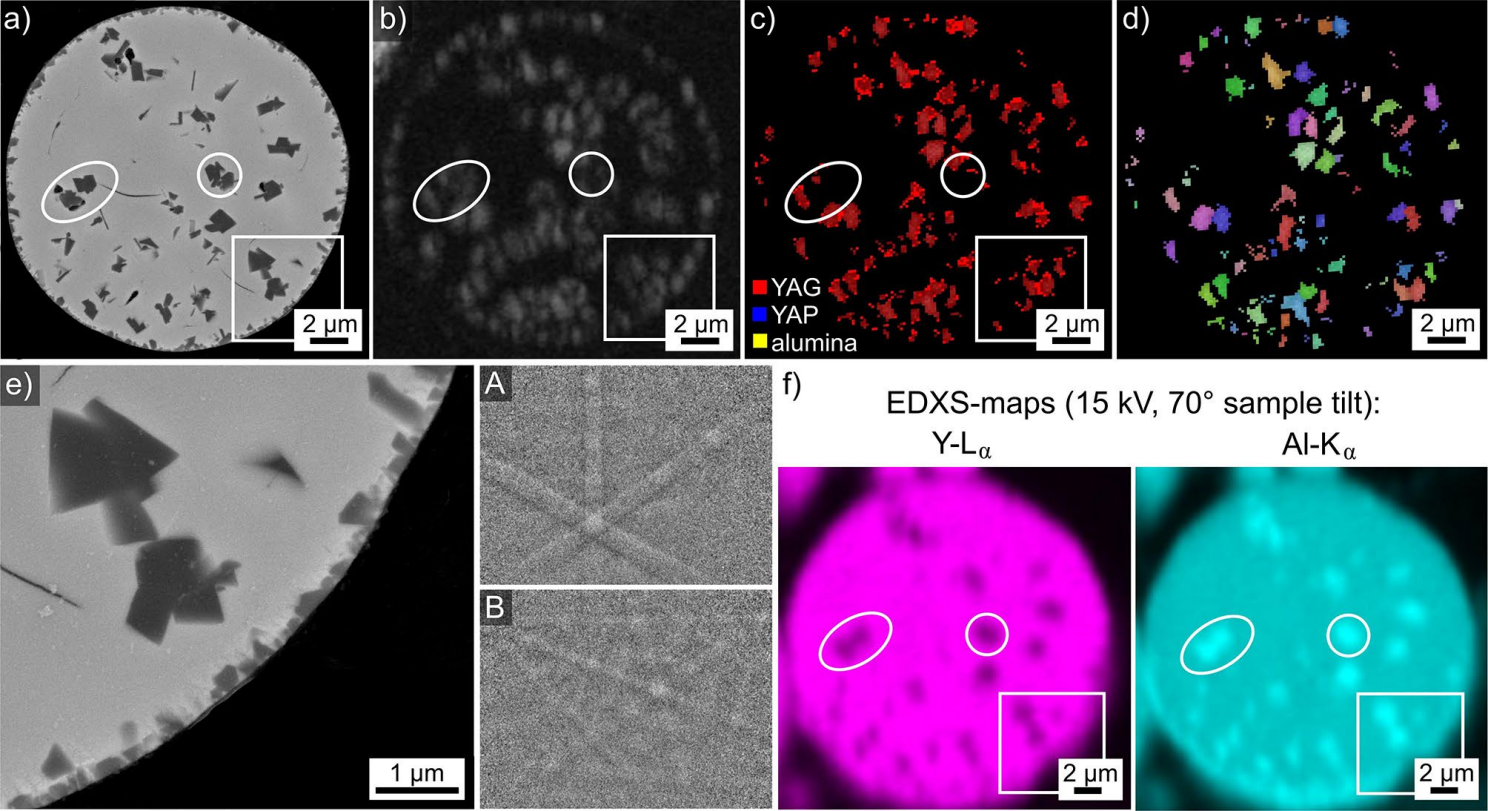
Results obtained from the microsphere type introduced in Fig. 2f: (**a**) SEM-micrograph of the microsphere and (**b**) BC-map of an EBSD-scan performed on the area. (**c**) phase + BC-map and (**d**) IPF + BC-map of the same area. The IPF-legend for cubic phases like YAG is presented in Fig. 1. The framed area is presented in greater detail (**e**) while (**f**) presents EDXS-maps of Y and Al. Selected areas are circled to ease comparability. The EBSD-pattern 8A was acquired from a structure appearing dark in the SEM-micrographs while pattern 8B was acquired from the area appearing bright.

Results obtained from the microsphere type introduced in Fig. 2f are presented in Fig. 8 where locations of specific interest have been circled in the SEM-micrograph (a) but also the BC-map (b) and BC + phase map (c) of an EBSD-scan performed on the area. Comparing the SEM-micrograph with the BC-map shows that EBSD-patterns could be acquired at far more locations than the dark areas in the SEM-micrograph. Furthermore, all indexed data points in Fig. 8c are attributed to YAG. The BC + IPF map of the area in Fig. 8d illustrates that these YAG-crystals have independent orientations throughout the sphere.

A closer look at the circled areas reveals that only some of the data points providing elevated values in the BC-map are indexed as YAG while others fail to be indexed and hence do not contribute to the BC + phase map. The latter usually originate from the locations of the dark structures in the SEM-micrograph. EBSD-pattern 8A was acquired from such a dark structure and fails to be indexed by the software, probably because it only contains one clear zone axis and is generally of poor quality. In contrast, pattern 8B was acquired from the brighter area in the sphere and is indexed as YAG. Pattern 8A contains significantly wider Kikuchi-Bands than pattern 8B. Considering the nominal composition of this sphere stated in Table 1, the dark contrast of these structures in the SEM-micrograph and the EBSD-pattern examples presented in Fig. 1, it is plausible to assume that pattern 8A originates from an aluminum oxide crystal.

The area framed in Fig. 8a is presented in greater detail in Fig. 8e to show that there is no discernible contrast amongst the bright areas in the SEM-micrograph while the BC- and BC + phase map of this area clearly show that some locations provide EBSD-patterns while others do not. Furthermore, it is clear that this microsphere shows a layer of surface crystallization apart from the easily discernible bulk crystallization. It is also confirmed that the dark crystals contribute contrast to the BC map but fail to provide indexable data points to the BC + phase map.

The EDXS-maps of Y and Al acquired along with the performed EBSD-scan show that the large, dark areas in the SEM-micrograph are enriched in Al but depleted of Y while no further contrast is discernible. The composition of the bright areas averaged over 5 EDXS-spot measurements acquired from the crystallized microsphere was measured to be 11.6 Y–31.6 Al–56.8 O at% and comparable values were acquired from neighboring uncrystallized spheres. The composition measured from the locations enriched in Al but depleted of Y in the EDXS-maps averaged over 5 EDXS-spot measurements is 2.2 Y–38.6 Al–59.2 O at%, basically matching the composition of Al_2_O_3_ when considering the information volume of EDXS, the crystallite size and the Y-containing matrix.

The presented results enable to conclude that this microsphere type is actually a three-phase system containing uncrystallized glass and YAG (undiscernible in the SEM-micrograph and EDXS-maps) as well as dark crystals which are very likely composed of an aluminum oxide. Similarly dark crystals of dendritic morphology also failed to provide indexable EBSD-patterns in a related glass where YAG crystallized at the surface^7^. They were subsequently confirmed to be composed of Al_2_O_3_ by transmission electron microscopy (TEM) analyses^8^. Their morphology in Fig. 8a is similar to that observed in Fig. 9c of Ref. ^18^ and, if related, the EBSD-pattern in Fig. 8A disproves the idea that these dark domains are amorphous^18^.

**Figure 9:**
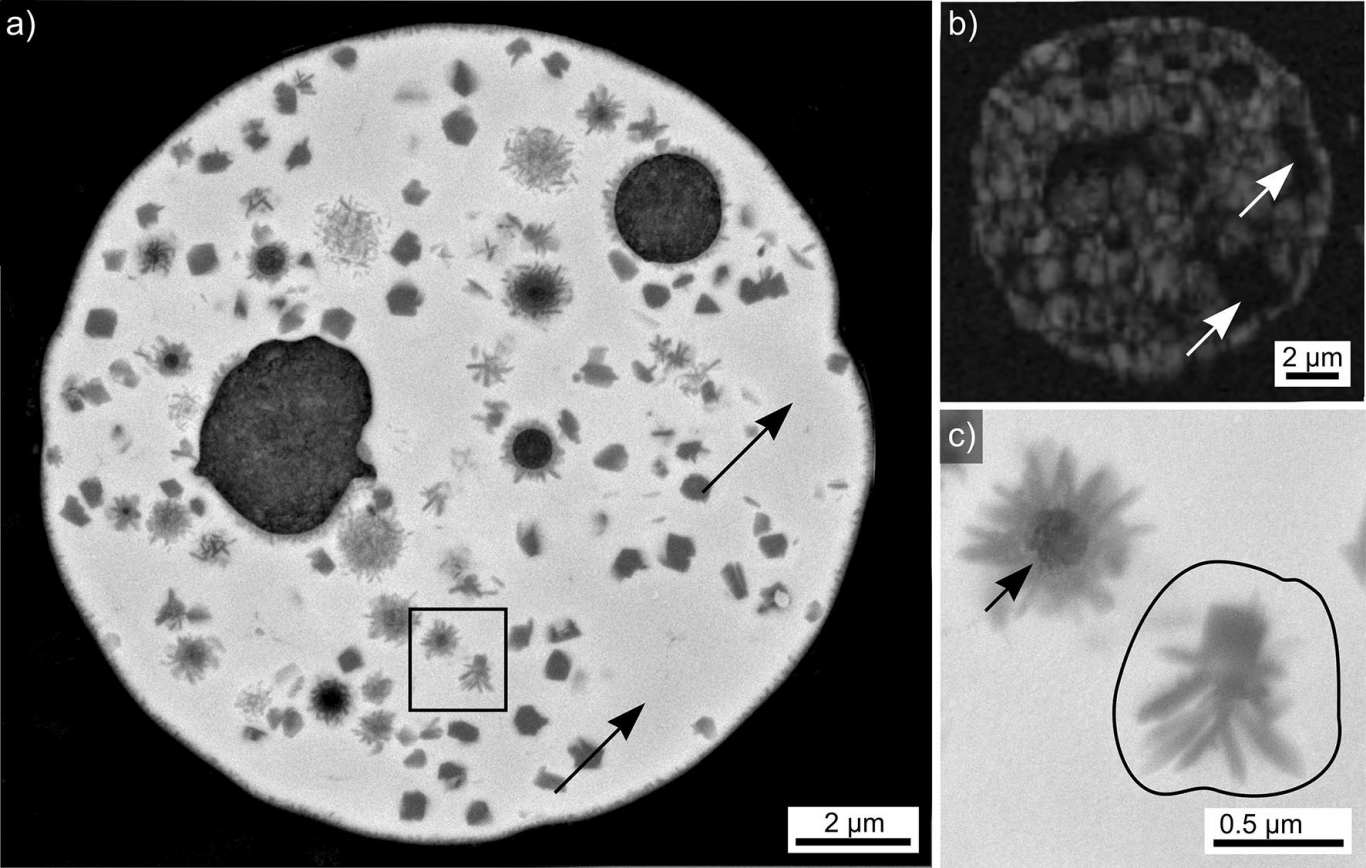
Results obtained from the microsphere type introduced in Fig. 2g: (**a**) SEM-micrograph, (**b**) BC map of an EBSD-scan performed on the area and (**c**) SEM-micrograph featuring the framed area in greater detail.

Considering the polygon morphology of the dark crystals as well as their high density in the outmost layer of surface crystallization, it is likely that the aluminum oxide nucleated first. As surface crystallization usually occurs before bulk nucleation, described in detail e.g. in the case of fresnoite^28^, the thin layer of dark crystals at the surface indicates that their growth into the bulk may have been blocked by the formation of YAG at the growth front, perhaps due to a local modification of the chemical composition caused by the subtraction of Al_2_O_3_ from the glass composition. The dark crystals in the bulk could have had more time to grow due to diffusion occurring in a 3D environment opposed to the 2D environment at the growth front of the surface crystallized layer.

Given the similarities in morphology and nominal composition, this microsphere type is probably the same as that presented in Fig. 9c of Ref. ^18^.

Results obtained from the microsphere type introduced in Fig. 2g are presented in Fig. 9. It basically provided comparable results as those presented in Fig. 8 with the difference that fine, dark structures of dendritic morphology occur in the SEM-micrograph of Fig. 9a next to the polygon structures. The BC map of an EBD-scan performed on this sphere in Fig. 9b indicates that it shows a much higher degree of crystallization than that presented in Fig. 8 although some areas highlighted by the arrows still fail to provide EBSD-patterns. All indexed data points are again attributed to YAG.

The area framed in Fig. 9a is presented in greater detail in Fig. 9c to show that the dendritic structures nucleate intensely at the surface of pores (arrow in Fig. 9c), a behavior also described for fine, dendritic aluminum oxide crystals observed in a related glass ^7,8^. The circled area in Fig. 9c is highly interesting because it shows a polygon crystal in direct contact with the dark, dendritic structures. While it is possible that the matrix-polygon interface served as a nucleation site for the dendrites similar to the pore surfaces, it is also possible that the polygon phase continued to grow via the dendritic mechanism due to a change of the local growth velocity. For example, YAG-crystals with the polygon morphology at one end but the dendritic morphology at another have been described at the boundary of a stria in a YAG-crystallizing glass^9^. Here the dendritic end of the crystals was located in a matrix containing less Si, i.e. probably with a higher viscosity at the same temperature, which would allow faster growth and the growth mechanism to change from dendritic to polygon growth^9^.

All in all, the presented results indicated the fine, dark dendritic structures are most probably composed of an aluminum oxide just like the polygons. Hence the dendritic structures described in Ref. ^20^ showing a very similar contrast and morphology are probably not composed of the stated YAG crystals. Instead, it is likely that the YAG crystals detected during HT-XRD analysis are not discernible in the presented SEM-micrographs^20^ similar to those presented in Figs. 8 and 9. Furthermore it should be noted that both phases probably nucleate at temperatures below the values where they were detected by XRD because the first crystals were not enough to produce a signal significant to the XRD-pattern.

**Figure 10:**
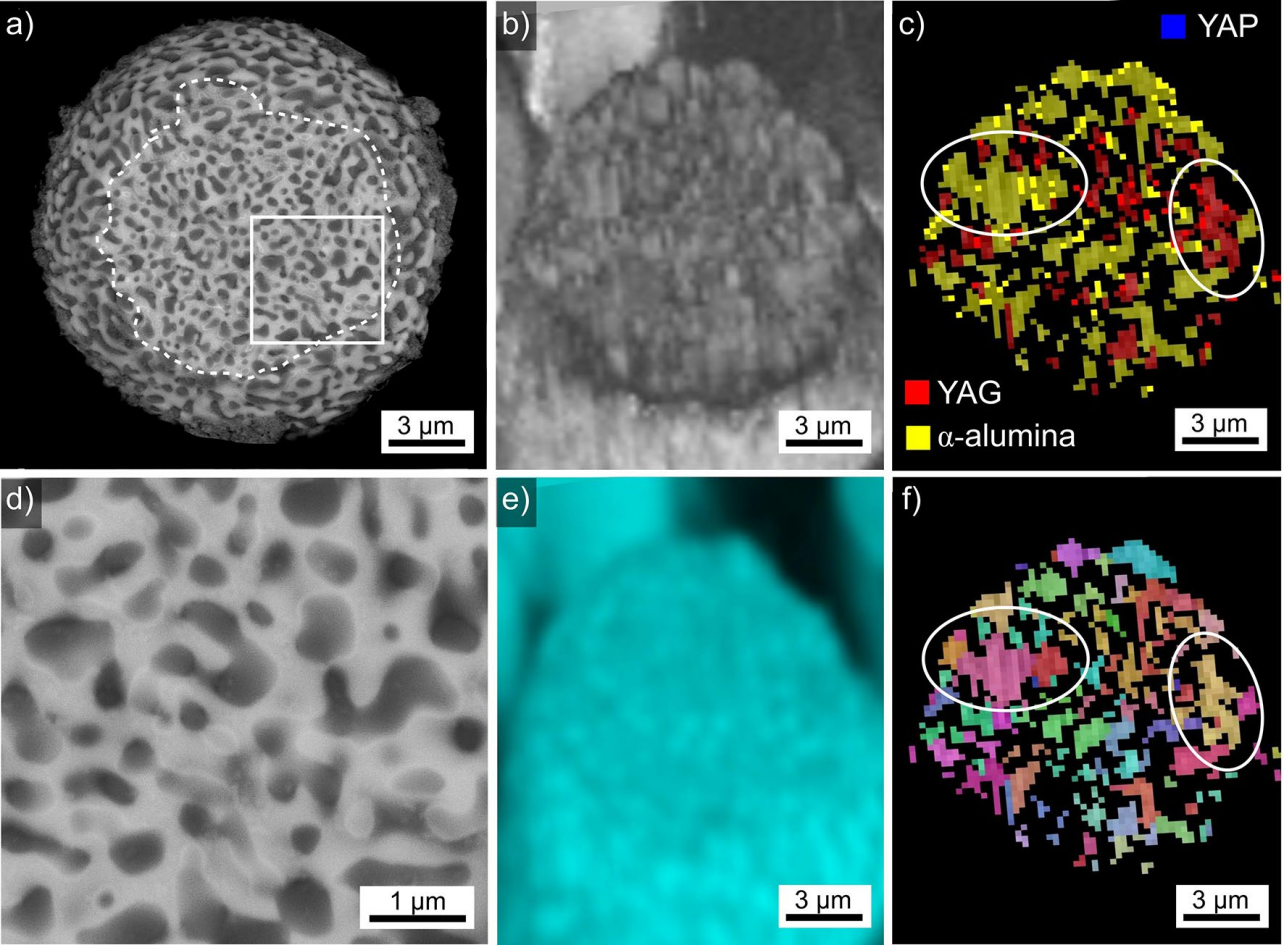
Results obtained from the microsphere type introduced in Fig. 2h: (**a**) SEM-micrograph where the dashed line outlines the polished cross section. (**b**) BC map of an EBSD-scan performed on the area. (**c**) Phase + BC map of the same scan. (**d**) Framed area in greater detail. (**e**) EDXS-element map of Al and (**f**) IPF + BC-map of the performed EBSD-scan. The IPF-legends for cubic and trigonal phases in Fig. 1 apply.

Results obtained from the microsphere type introduced in Fig. 2h are presented in Fig. 10. This microsphere was partially exposed from the embedding material during preparation to that the plane of the cross section, circled by the dashed white line in the SEM-micrograph Fig. 10a, only transects part of the visible sphere. It contains areas of a dark and bright contrast in the SEM-micrograph, both usually showing diameters of less than 1 µm. The BC map of an EBSD-scan performed on this area is presented in Fig. 10b while c presents the corresponding BC + phase map, illustrating that YAG and α-alumina are detected throughout this sphere while YAP is not observed. Two areas have been circled to show that the regions attributed to either phase are frequently far larger than the phase domains discernible in the SEM-micrograph. The area framed in Fig. 10a is presented in greater detail in Fig. 10d to show that this microstructure contains the rounded droplets and channels associated with phase separation rather that the morphologies known to originate from crystal growth. Figure 10e features an EDXS-map of Al acquired during the performed EBSD-scan to show that only minimal contrast is discernible due to the small size of the phase domains and the information volume of EDXS. The composition of this microsphere averaged over 10 EDXS-spot measurements (5 from dark and 5 from the bright areas) was measured to be 9.4 Y–34.7 Al–55.9 O at%. Evaluating these phases separately is useless because the deviation within the points of each measured group is larger than between the groups, verifying that these phase domains are simply too small for reliable EDXS-analyses in the SEM.

Finally, the IPF + BC map of the performed EBSD-scan is presented in Fig. 10f to show that the areas circled in Fig. 10c also show a relatively homogeneous orientation. This means that the phase components in this sphere are connected outside of the current cross section. It may be concluded that the glass in this sphere first phase separated and subsequently crystallized to form interpenetrating grains of YAG and α-alumina. As the separated amorphous phases apparently did not manage to minimize their surface by forming independent droplets, the microstructure of interpenetrating channels must have been stabilized by either a significant increase of the viscosity in one of the liquids or an almost immediate crystallization. The comparably large orientation domains discernible in Fig. 10f indicate the almost immediate crystallization to be more probable. Any relation to the density driven phase separation observed in the Y_2_O_3_–Al_2_O_3_ system after very high undercooling temperatures^5^ is very unlikely as the phase separated liquids observed here allowed the crystallization of clearly differing crystal phases with different densities.

Given the similarities in morphology and nominal composition, this microsphere type is probably the same as that presented in Fig. 9f of Ref. ^18^. It is also noteworthy that a similar microstructure with similarly connected orientation domains was observed in ceramics produced from glass microspheres^21^.

**Figure 11:**
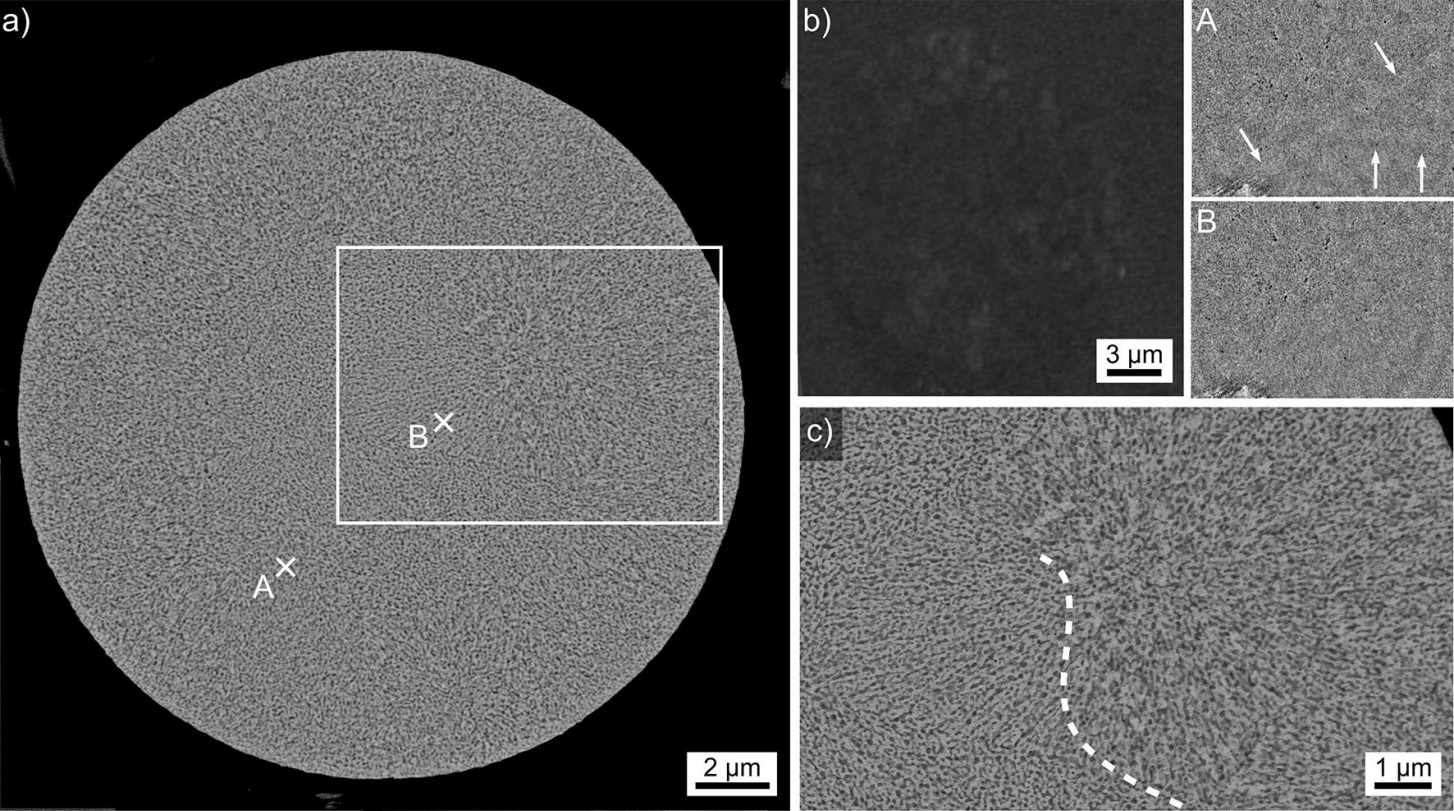
Results obtained from the microsphere type introduced in Fig. 2j: (**a**) SEM-micrograph of the microsphere and (**b**) BC map of an EBSD-scan performed on the area. The EBSD-patterns 11A and 11B were acquired at the locations A and B, white arrows indicate weak Kikuchi-bands. (**c**) SEM-micrograph presented in the framed area in greater detail, the dashed line highlights the boundary between the surface area and the bulk.

The microsphere type introduced in Fig. 2j was analyzed in detail and the results are summarized in Fig. 11. The SEM-micrograph in Fig. 11a shows little contrast and the EBSD-patterns 11A and 11B illustrated the poor pattern quality obtained from this sphere. None of the patterns acquired from it could be indexed for lack of pattern quality. However, the weak Kikuchi-bands highlighted in pattern 11A prove that there is a crystal lattice within the information depth of EBSD which should be limited to less than 36 nm for the measurements performed here^27^. Similar results have been obtained from very fine crystals in a SiO_2_/Al_2_O_3_/Y_2_O_3_/AlF_3_/B_2_O_3_/Na_2_O glass-ceramic^7^ which were later confirmed to be Al_2_O_3_ using TEM^8^. On the other hand, pattern 11B acquired at a location closer to the bulk shows no discernible Kikuchi bands at all.

The BC map of an EBSD-scan performed on this sphere is presented in Fig. 11b. It was acquired using a binning of 2 × 2 and shows some barely discernible regions of elevated band contrast around the periphery of the sphere, indicating surface crystallization. Figure 11c features the framed area in greater detail to illustrate that the microstructure in this microsphere is composed of two phases interpenetrating each other with domain sizes in the nm-scale. The dashed line illustrates the boundary between the probably surface crystallized layer and the bulk.

Such microstructures have been described to originate from phase separation^30,31^ and problems acquiring EBSD-patterns from it have been reported for surface crystallizing Ba-fresnoite crystals growing in phase separating glasses^30,31^. The composition of this sphere type averaged over 5 EDXS-spot measurements acquired from this microsphere was measured to be 10.4 Y–32.5 Al–57.1 O at%. However, given the information volume of EDXS and the microstructure of the sphere, this composition represents the average composition of both phases occurring after phase separation.

**Figure 12:**
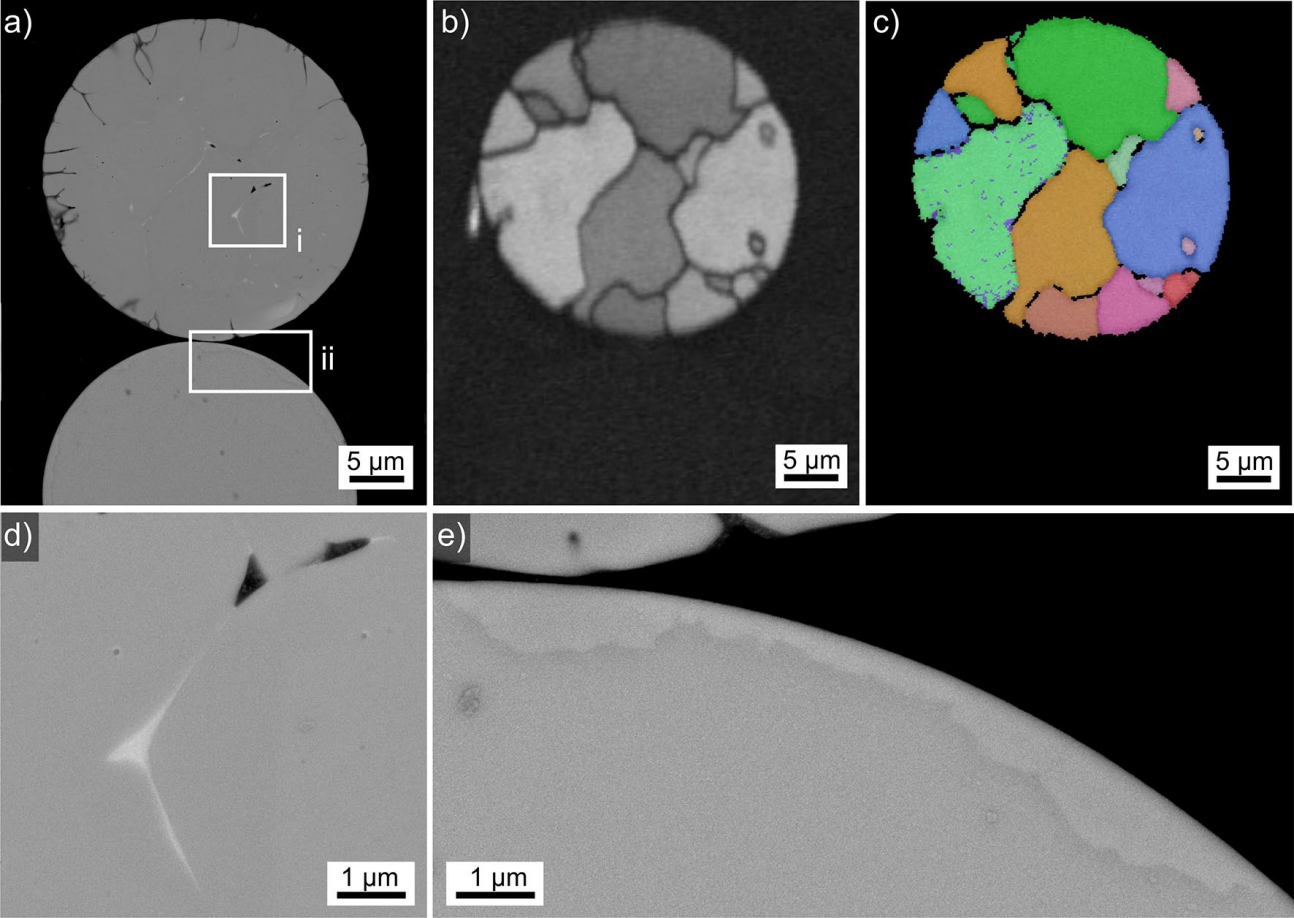
Results obtained from the microsphere type introduced in Fig. 2k: (**a**) SEM-micrograph of the microsphere. (**b**) BC-map and (**c**) IPF + BC-map of an EBSD-scan performed on the microsphere, same legend as in Fig. 16c. (**d**) Area in frame (i) in greater detail and (**e**) the area in frame (ii) in greater detail.

Results obtained from the microsphere type introduced in Fig. 2k are presented in Fig. 12, all EBSD-patterns acquired from the analyzed area were indexed as YAG. Of the two microspheres featured in Fig. 12a, only the top one provided EBSD-patterns as illustrated by the BC map in Fig. 12b of an EBSD-scan performed on the area. The BC + IPF map in Fig. 12c shows that the sphere only contains a few large grains, orientation relationships amongst them are not indicated. However, the same orientations occur at various locations of the sphere, i.e. pale green and orange, indicating these crystal lattices are probably connected outside of the current cross section. Hence the grains are probably even larger than indicated by the grain boundaries in the current cross section, but dendritic growth is not indicated by the crystal morphology.

The area in frame (i) is presented in greater detail in Fig. 12d to show that a secondary phase (bright contrast, i.e. probably enriched in Y) and some pores (dark contrast) occur at the grain boundaries. While the bottom sphere in Fig. 12a failed to provide any significant signal for EBSD-analysis, Fig. 12e shows that there is a thin layer of what is probably surface crystallization, i.e. this sphere was just beginning to crystallize before it was cooled and subsequently analyzed. The composition of this sphere type averaged over 5 EDXS-spot measurements acquired from the crystallized microsphere was measured to be 18.1 Y–26.9 Al–55.0 O at%, comparable values were acquired from neighboring uncrystallized spheres.

Given the similar morphology of this microsphere type with that depicted in Fig. 9b of Ref. ^18^ and Fig. 8 of Ref. ^19^, it is likely that Fig. 12 illustrates the microstructure of the spheres discussed in Ref. ^19^.

**Figure 13:**
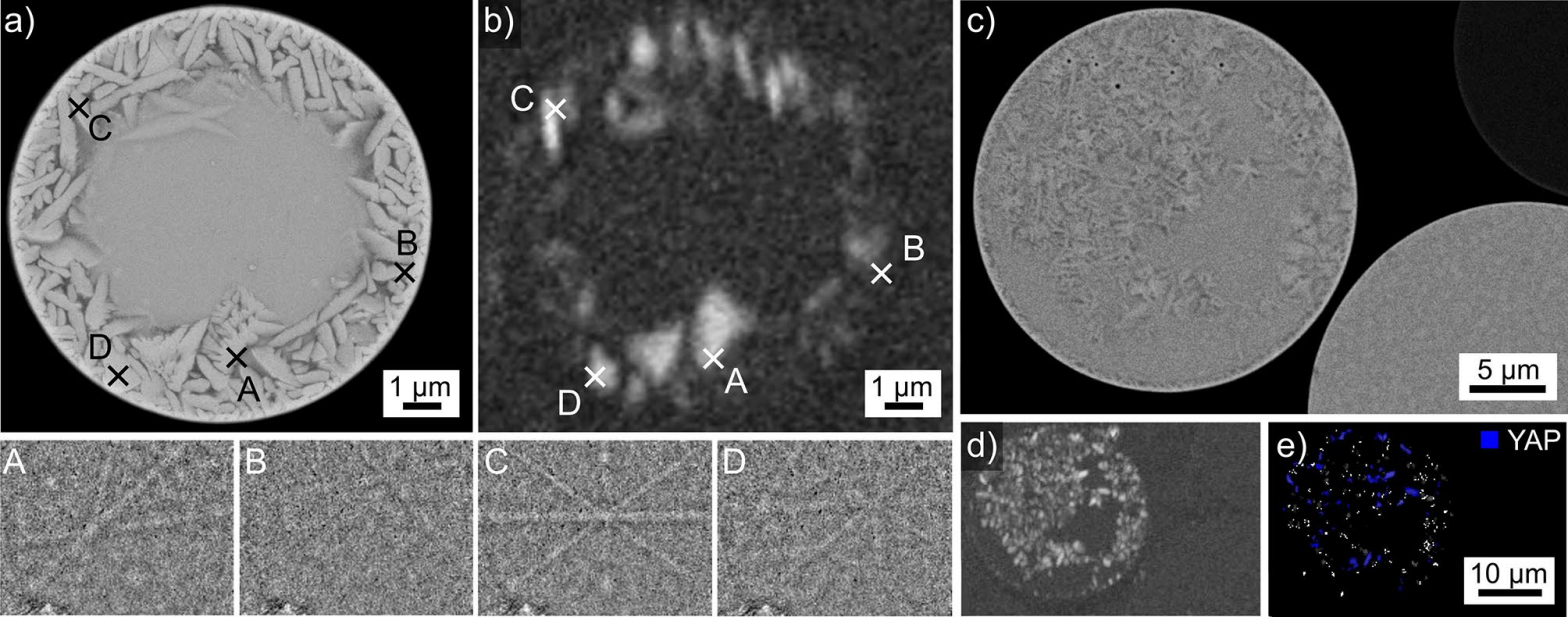
Results obtained from the microsphere type introduced in Fig. 2L: (**a**) SEM-micrograph of the microsphere and (**b**) BC map. The EBSD-patterns 13A–13D were acquired at the locations A–D. (**c**) SEM-micrograph featuring three related microspheres and (**d**) BC map and (**e**) BC + phase map of an EBSD-scan performed on the area.

Results obtained from the microsphere type introduced in Fig. 2L are presented in Fig. 13 where the SEM-micrograph (a) clearly indicates surface crystallization and rudimentary dendritic growth morphologies are discernible. The BC-map of an EBSD-scan performed on this sphere is presented in Fig. 13b to show the EBSD-pattern acquisition is possible but inhomogeneous within the surface crystallized layer. The EBSD-patterns 13A-13D were acquired at the locations A-D and illustrated the poor pattern quality often obtained from the sphere: while the patterns 13A and 13B are indexed as YAP, the patterns 13C and 13D failed to be indexed as either YAP or YAG. α-alumina and YAM can be excluded as both would show a darker contrast in the SEM-micrograph.

Figure 13c highlights a larger microsphere subjected to the identical thermal treatment: it clearly shows surface as well as bulk nucleation. Figure 13c also contains segments of neighboring spheres: a sphere of darker contrast (top right) and a sphere of bright contrast showing heterogeneities indicating crystallization but without clear dendritic morphologies. The BC map of an EBSD-scan performed on this area is presented in Fig. 13d and shows that only the large sphere enabled the acquisition of EBSD-patterns. The BC + phase map of the same scan is presented in Fig. 13e and shows that all reliably indexed data points in this sphere were indexed as YAP.

The chemical composition of bright, crystallizing sphere type in Fig. 13 averaged over 10 EDXS-spot measurements acquired from different microspheres was measured to be 24.9 Y–20.0 Al–55.1 O at%. A significant difference between crystallized and uncrystallized regions could not be measured. The composition of the darker sphere featured in Fig. 13 c) averaged over 5 EDXS-spot measurements was measured to be 10.8 Y–32.9 Al–56.3 O at%.

**Figure 14:**
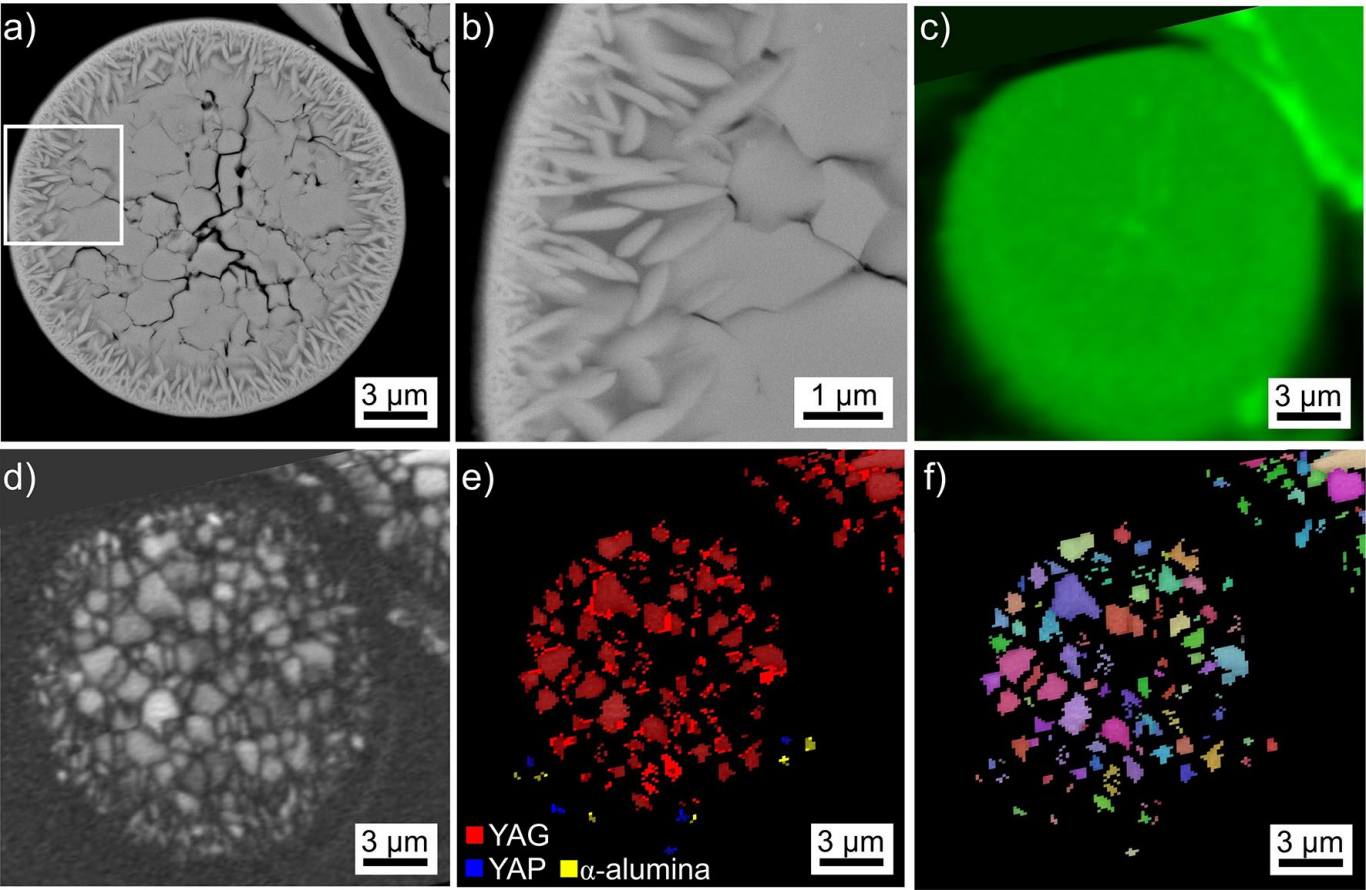
Results obtained from the microsphere type introduced in Fig. 2m: (**a**) SEM-micrograph and (**b**) the framed area in greater detail. (**c**) An element map of Al acquired by EDXS during an EBSD-scan performed on the area. (**d**) BC map, (**e**) BC + phase map and (**f**) BC + IPF map of the same EBSD-scan. The IPF-legends for cubic, orthorhombic and trigonal phases in Fig. 1 apply.

The microsphere introduced in Fig. 2m and also presented in Fig. 14a shows a similar layer of surface crystallization as that discernible in Fig. 13a, but here bulk crystallization with significantly larger grains is also indicated. The more detailed micrograph of the framed area presented in Fig. 14b shows that the grain size in the surface layer increased during growth, indicating a kinetic selection. A transition from the surface layer to the bulk microstructure is not observed, instead the bulk grains seem to adjust to the morphology of the finer surface crystals. This indicates the surface layer was already present when the bulk crystallized which is in agreement with the observation that surface crystallization generally occurs before bulk crystallization.

The element map of Al in Fig. 14c fails to show a significant contrast between the surface layer and the bulk. EDXS-spot measurements acquired from the surface and bulk crystallized regions also failed to show any significant compositional difference and the composition averaged over 10 spot measurements of this sphere is 19.7 Y–27.2 Al–53.1 O at%. The BC map of the area presented in Fig. 14d shows that the pattern quality obtained from the small crystals in the surface layer is lower than that obtained in the bulk. The BC + phase map of the performed EBSD-scan in Fig. 14e shows that the bulk crystals are composed of YAG while only a few indexable data points attributed to either YAP or α-alumina were obtained from the surface crystallized region. The BC + IPF-map in Fig. 14f only shows independent orientations of the crystals measured in this microsphere.

The results presented above allow the conclusion that the surface crystallized layer probably containing YAP and maybe α-alumina formed in the melt and its growth was stopped by the quenching procedure. The large YAG grains in the bulk, however, probably formed during the subsequent heat treatment which is why they adapt to the morphology of the already present layer of surface crystallization.

**Figure 15:**
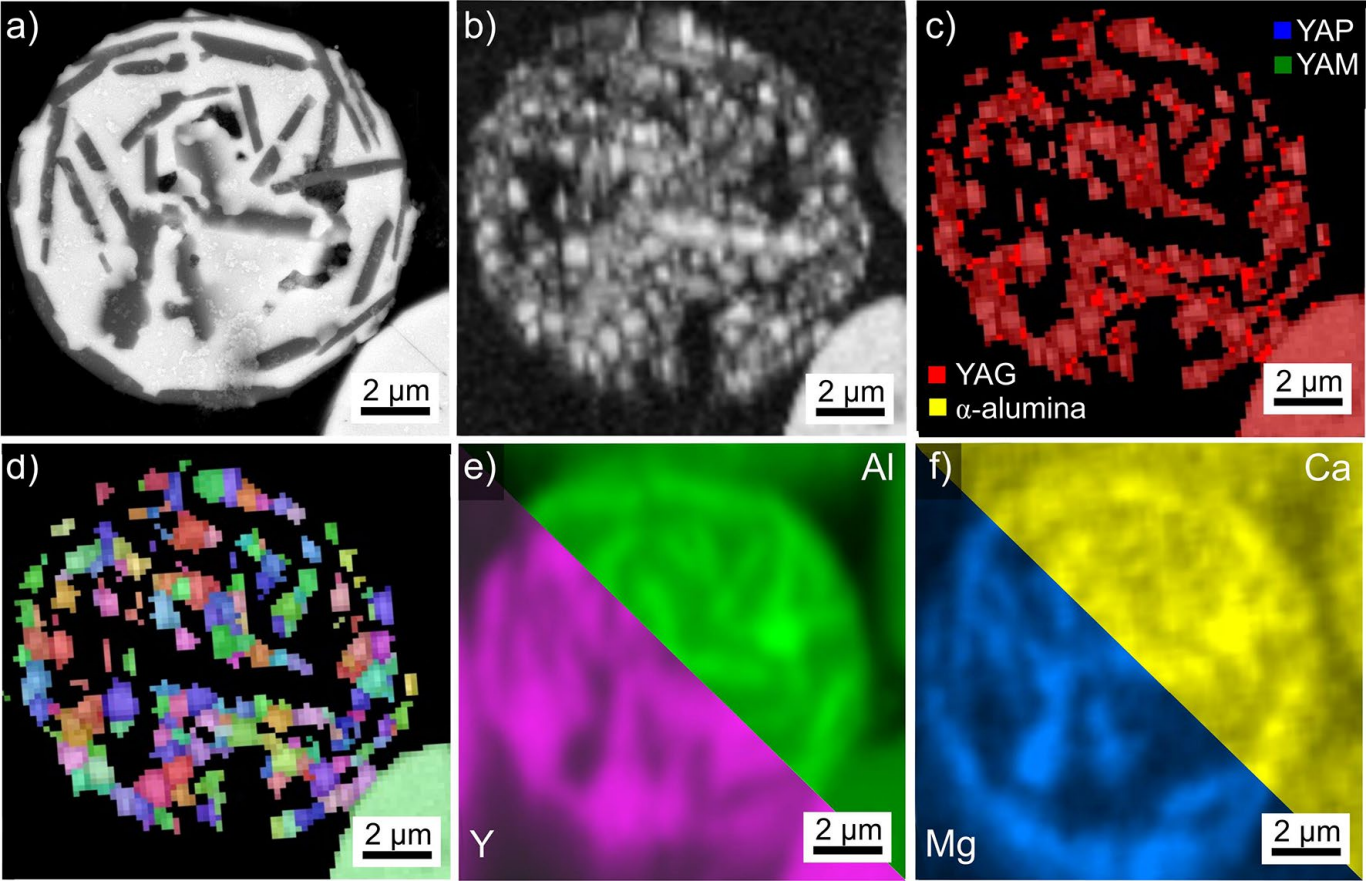
Results obtained from the microsphere type introduced in Fig. 2n: (**a**) SEM-micrograph and (**b**) BC map of an EBSD-scan performed on the surface. (**c**) The BC + phase map and (**d**) the BC + IPF map of the same scan. The IPF-legends for cubic, orthorhombic and trigonal phases in Fig. 1 apply. Partial element maps of (**e**) Y and Al and (**f**) Mg and Ca acquired during the EBSD scan are presented.

The microsphere introduced in Fig. 2n is also presented in Fig. 15a and shows straight, dark structures indicating crystal plates embedded in a bright matrix. Interestingly, there is a thin layer of these dark plates parallel to the outer boundary of the microsphere, indicating surface nucleation of this phase with some orientation preferably aligned to the surface. Oriented nucleation has been proven in a number of surface crystallizing glasses, a short summary was recently presented in the context of a review concerning fresnoite glass–ceramics^28^. Sadly, analyzing the immediate surface of such a microsphere by EBSD in the current context is not feasible: even if such a microsphere could be located without embedding, the size and surface curvature of the sphere would make any representative texture analysis impossible.

The BC-map in Fig. 15b of an EBSD-scan performed on this sphere shows that EBSD-patterns were acquired throughout the sphere, however, the phase map in Fig. 15c shows that the patterns from the dark plates fail to be indexed: all indexed data points in the scan are attributed to YAG. The BC + IPF-map in Fig. 15d shows that YAG form independent grains in this microsphere.

EDXS-spot measurements performed on this sphere showed contaminants of Ca and Mg not observed in neighboring spheres. The chemical composition of bright areas attributed to YAG in the phase map in Fig. 15 averaged over 5 EDXS-spot measurements was measured to be 13.0 Y–31.4 Al–54.7 O + 0.2 Mg and 0.7 Ca in at%, confirming a composition close to YAG. By contrast, the dark crystals showed a composition of 5.4 Y–35.4 Al–56.9 O + 0.6 Mg and 1.7 Ca in at% also averaged over 5 EDXS-spot measurements, supporting the hypothesis that these crystals probably contain an aluminum oxide crystal lattice.

The partial element maps of Y and Al in Fig. 15e match the result that Al is enriched in the dark structures while Y is depleted. In contrast, the partial element maps of Mg and Ca in Fig. 15f show that both elements are enriched in the dark crystals. As both Mg and Ca should function as network modifiers and hence lower melting points and viscosities of glasses in the analyzed system, it is plausible to assume that these contaminants furthered the crystallization of this sphere.

Both Ca and Mg are known as sintering aids and dopants for YAG^36,37^ and alumina^38–40^. In the case of alumina ceramics, both Mg and Ca preferably accumulate at the grain boundaries instead of in the alumina grains^40^, hence it is unlikely that the amounts detected by EDXS here are really incorporated into the aluminum oxide lattice. It is plausible to assume that some segregation of these elements could cause a distortion of the aluminum oxide crystal lattice which could prevent the EBSD-patterns from being indexed as α-alumina by the software.

**Figure 16:**
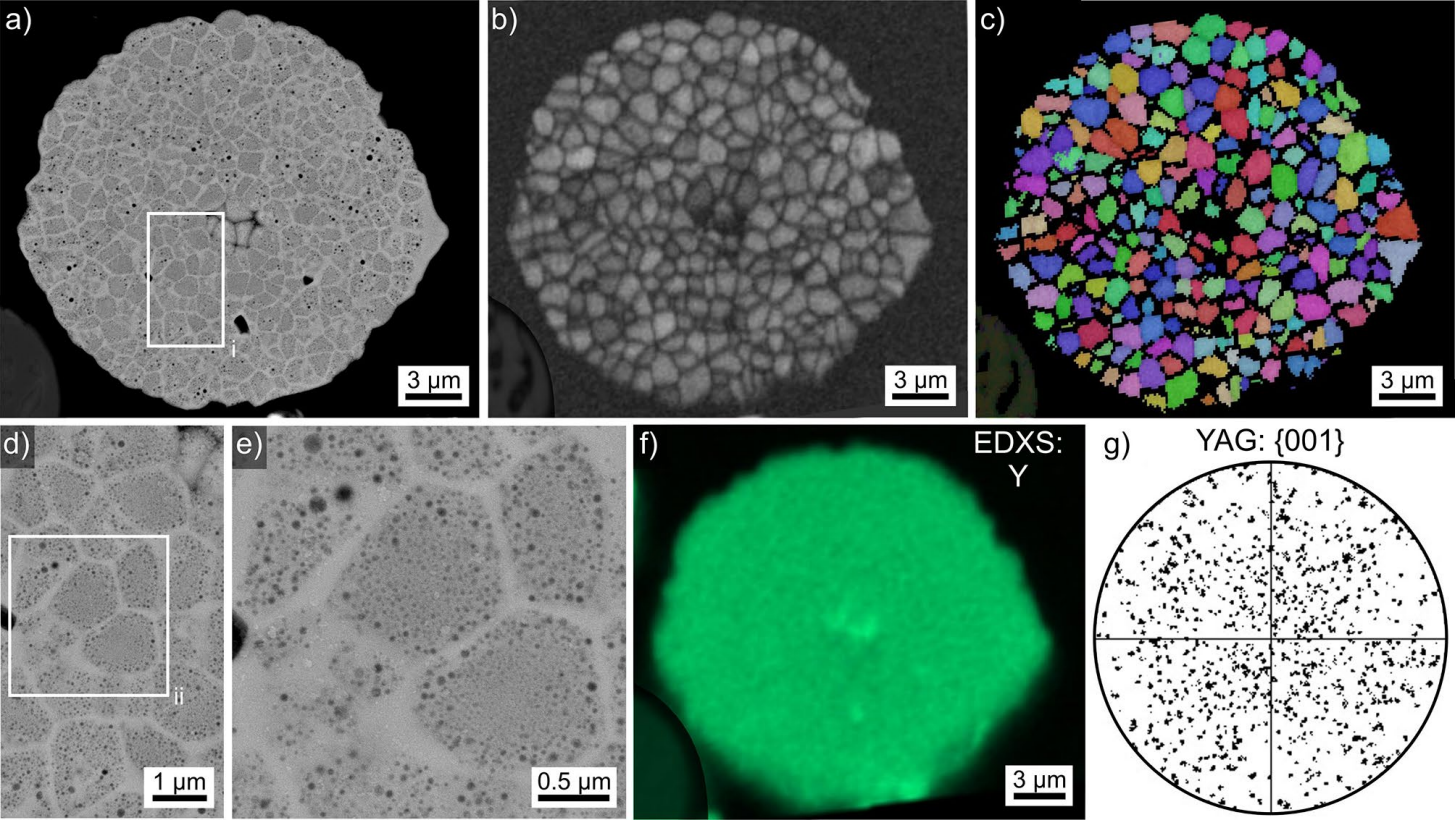
Results obtained from the microsphere type introduced in Fig. 2o: (**a**) SEM-micrograph of the microsphere. (**b**) BC-map and (**c**) IPF + BC-map of an EBSD-scan performed on the microsphere. The IPF-legend for cubic phases in Fig. 1 apply. The SEM-micrograph (**d**) presents the area in frame (i) in greater detail while the area in frame (ii) is featured in the SEM-micrograph (**e**). (**f**) An element map of Y collected by EDXS during the EBSD-scan and (**g**) the {001} PF of all data points indexed as YAG.

Results obtained from the microsphere type introduced in Fig. 2o are presented in Fig. 16, all EBSD-patterns acquired from this sphere were indexed as YAG. While the SEM-micrograph overview (a) shows grains and pores in this microsphere, the BC map of an EBSD-scan performed on it provides a clearer impression of the grain structure but fails to show most of the pores due to the limited resolution (step size 150 nm). While the grain boundaries appear brighter in the SEM-micrographs indicating a locally elevated backscatter coefficient, the grain boundaries are darkened in the BC map which is often caused by a less perfect crystal lattice near grain boundaries as well as EBSD-pattern superposition caused by the presence of multiple orientation domains occurring within the significant information volume of EBSD^27^. The BC + IPF map of the performed scan presented in Fig. 16c shows that there are no orientation relationships between the individual grains, pointing towards an homogeneous crystal nucleation throughout the microsphere followed by relatively slow crystal growth via either spherical or polygon growth until the grains collide.

A closer look at the microstructure in frame (i) is presented in Fig. 16d and further insight is provided by Fig. 16e detailing the area in frame (ii): the grains appearing to be homogenous in the EBSD-results are in fact composed of at least two phases. One of these occurs in the form of dark droplets which show larger diameters near the grain boundaries at which they do not occur. Most probably the secondary phase currently accumulated in the droplets diffused away from the grain boundaries and was added to nearby droplets, allowing them to grow. As the grains are systematically attributed to YAG, it is probable that these droplets contain residual glass composed of the elements not included into the YAG crystal lattice.

The composition of this sphere type averaged over 9 EDXS-spot measurements acquired from different microspheres was measured to be 15.6 Y–28.7 Al–55.7 O at% and traces of Fe amounting to less than 0.1 at%. This is close to the theoretical composition of YAG. An EDXS-map acquired during the EBSD-scan failed to show any significant contrast as illustrated by the element map of Y presented in Fig. 16f. The {001} pole figure (PF) of all data points attributed to YAG in the EBSD-scan indicates a random orientation distribution, i.e. a texture is not discernible in the analyzed cross section.

**Figure 17:**
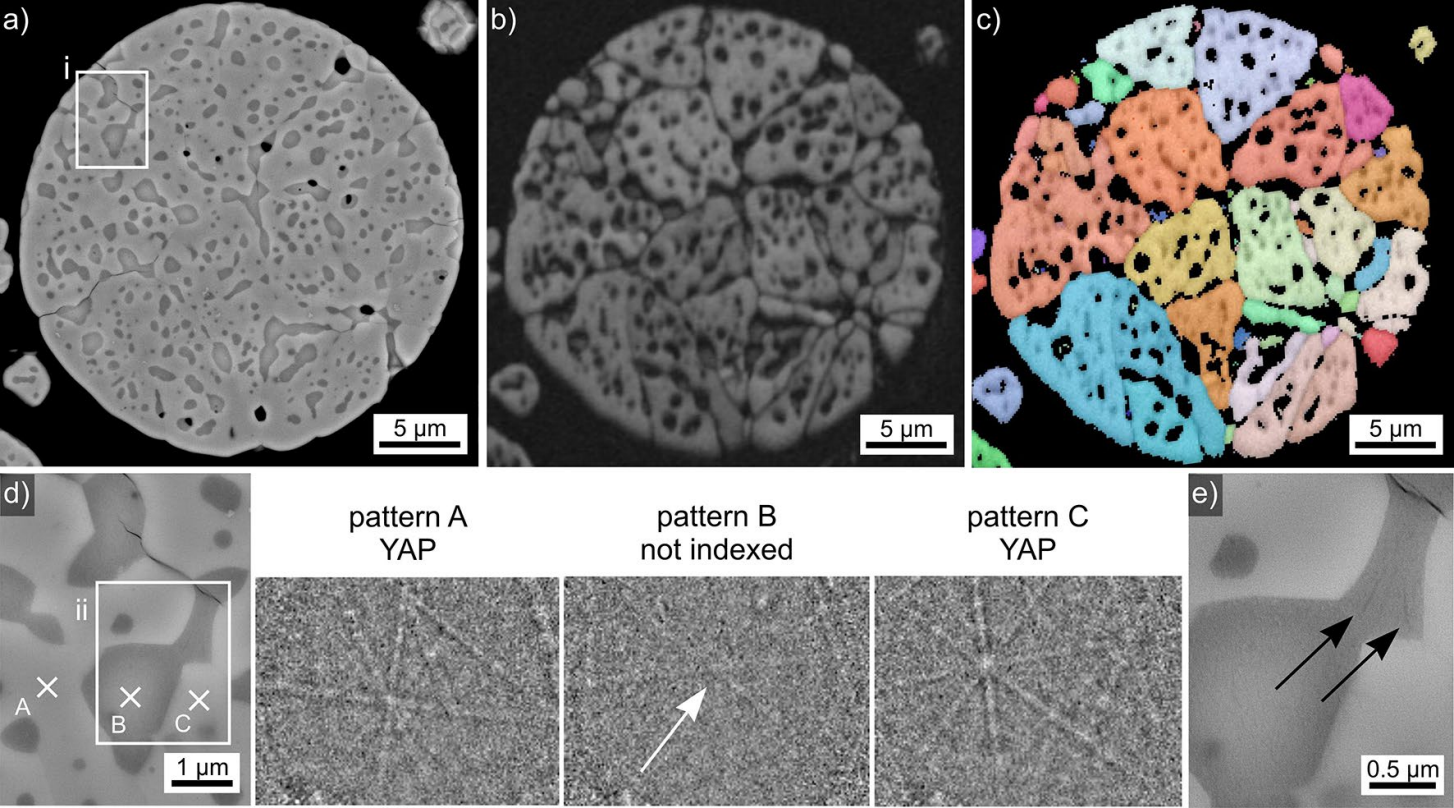
Results obtained from the microsphere type introduced in Fig. 2p: (**a**) SEM-micrograph of the microsphere. (**b**) BC-map and (**c**) IPF + BC-map of an EBSD-scan performed on the microsphere. The IPF-legend for orthorhombic phases in Fig. 1 apply. (**d**) Area in frame (i) in greater detail and (**e**) the area in frame (ii) in greater detail. The EBSD-patterns 17A–17C were acquired at the locations A–C.

Results obtained from the microsphere type introduced in Fig. 2p are presented in Fig. 17, all EBSD-patterns acquired from the analyzed area were indexed as YAP. The SEM-micrograph overview in Fig. 17a illustrates that this microsphere is composed of at least two phases which occur in the µm-scale. A clear grain structure is not discernible and the topography contrast proves that the phase of slightly darker contrast (secondary phase) was more sensitive to the applied polishing procedure as it systematically forms depressions in the sphere. The BC map of the EBSD-scan performed on it is presented in Fig. 17b and enables to discern a grain structure. A systematically lower BC of the secondary phase is discernible, indexable patterns were not acquired from it. The combined BC + IPF map of the EBSD scan presented in Fig. 17c shows that the secondary phase occurs at grain boundaries as well as inside the grains. There are no orientation relationships between the individual grains, pointing towards a homogeneous crystal nucleation throughout the microsphere followed by relatively slow crystal growth via either spherical or polygon growth until the grains collide. Due to the large size of the secondary phase and its rounded, sometimes elongated like morphology, the microsphere probably also showed phase separation before crystallization.

A closer look at the microstructure in frame (i) is presented in Fig. 17d to detail the locations A-C where the EBSD-patterns 17A-17C were acquired. While the patterns 17A and 17C are indexed as YAG, pattern 17B fails to be indexed, most probably due to its very poor quality. However, pattern 17B does contain a few very weak Kikuchi bands proving electron diffraction and the zone axis highlighted by the white arrow is not discernible in the patterns 17A and 17C originating from the neighboring crystal lattices. While this cannot be considered to prove crystalline components in the secondary phase considering the full information depth significant to EBSD^27^, the more detailed micrograph of the area in frame (ii) presented in Fig. 17e contains some heterogeneities inside the area attributed to the secondary phase, i.e. this could in fact contain a crystal lattice disturbed by residual glass.

The composition of the primary YAP crystals in this microsphere averaged over 5 EDXS-spot measurements using an acceleration voltage of only 6 kV to minimize the information volume was measured to be 27.6 Y–17.3 Al–55.1 O at% (neglecting traces of Fe amounting to less than 0.1 at%). Hence these YAP-crystals seem to be enriched in Y and depleted of Al given the theoretical at% composition of YAP (20 Y–20 Al–60 O). The comparably measured composition for the secondary phase is 25.5 Y–19.3 Al–55.2 O at%, i.e. it is possible to state that it contains less Y and more Al then the YAP-crystals even though the relatively large information volume of EDXS should be taken into account when considering these values.

**Figure 18:**
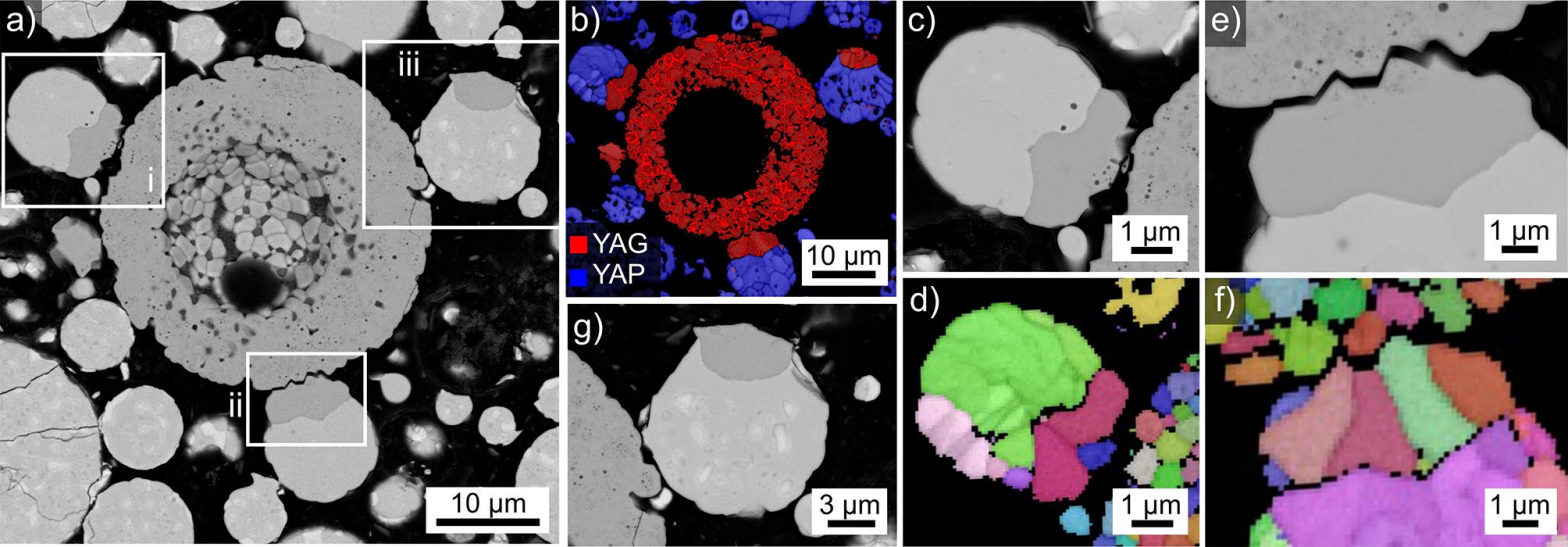
Contact of the microsphere types detailed in Figs. 16 and 17: (**a**) SEM-micrograph featuring a large (dark) microsphere surrounded by multiple smaller microspheres. (**b**) BC + phase map of an EBSD-scan performed on the area. (**c**) Area in frame (i) in greater detail. (**d**) BC + IPF map of the area in (**c**). The IPF-legend for cubic and orthorhombic phases in Fig. 1 apply. (**e**) Area in frame (ii) in greater detail. (**f**) IPF + BC-map of the area in (**e**). (**g**) Area in frame (iii) in greater detail. The IPF-legend of YAG is presented in Fig. 16 while that of YAP is presented in Fig. 17.

The microspheres featured in Figs. 16 and 17 were observed after the same production process. While the YAP microspheres detailed in Fig. 17 occurred far more often, they appear to transform to YAG when in contact to the YAG microspheres detailed in Fig. 16. This process is presented in detail in Fig. 18: a large microsphere of the type detailed in Fig. 16 surrounded by microspheres of the type detailed in Fig. 17 featured in the SEM-micrograph is presented in Fig. 18a. The phase + BC map of an EBSD-scan performed on this area is shown in Fig. 18b and confirms they respectively contain YAG and YAP. However, some of the YAP-microspheres contain areas attributed to YAG which predominantly occur where the YAP-spheres seem to contact the YAG-sphere, three of them are subsequently presented in greater detail.

The area in frame (i) is presented in greater detail in Fig. 18c to show that the area attributed to YAG (darker contrast) contains almost none of the droplets observed so frequently in the YAG-microsphere. The BC + IPF map of the EBSD-scan performed on this area is presented in Fig. 18d to visualize that only three grains attributed to YAG occur in this area, two of which are droplet free while the third (small) grain shows the same droplets also observed in the large YAG-microsphere featured in Fig. 16. It is hence likely that the droplet-containing grain is actually a segment of a YAG grain in the larger microsphere that broke off when the spheres were separated during sample preparation. The area in frame (ii) detailed in Fig. 18e presents similar results, only here the crack pattern at the boundary between the microspheres clearly implies they used to be connected. The grains illustrated in the corresponding BC + IPFmap in Fig. 18f show grain boundaries more or less perpendicular to this crack, supporting the concept that the transformation to YAG in the smaller microsphere is triggered by the contact to the larger YAG microsphere and then proceeds through the smaller YAP sphere.

Figure 18g shows the area in frame (ii) in greater detail. Here the YAG-domain in the small sphere is not near the large YAG sphere and there is no indication of a transformation to YAG near the interface. However, the interface does not imply any previous connection, so the most likely explanation for this observation that the small sphere has no physical contact to the large YAG-sphere featured in Fig. 18a. Instead, it may be in contact to a different YAG-sphere outside of the current cross section or it may have been separated from a YAG-sphere during sample preparation. The results presented here indicate that the statement that “…fully or partially crystallized glass microspheres do not influence crystallization of other beads”^20^ may not apply to all cases.

**Figure 19:**
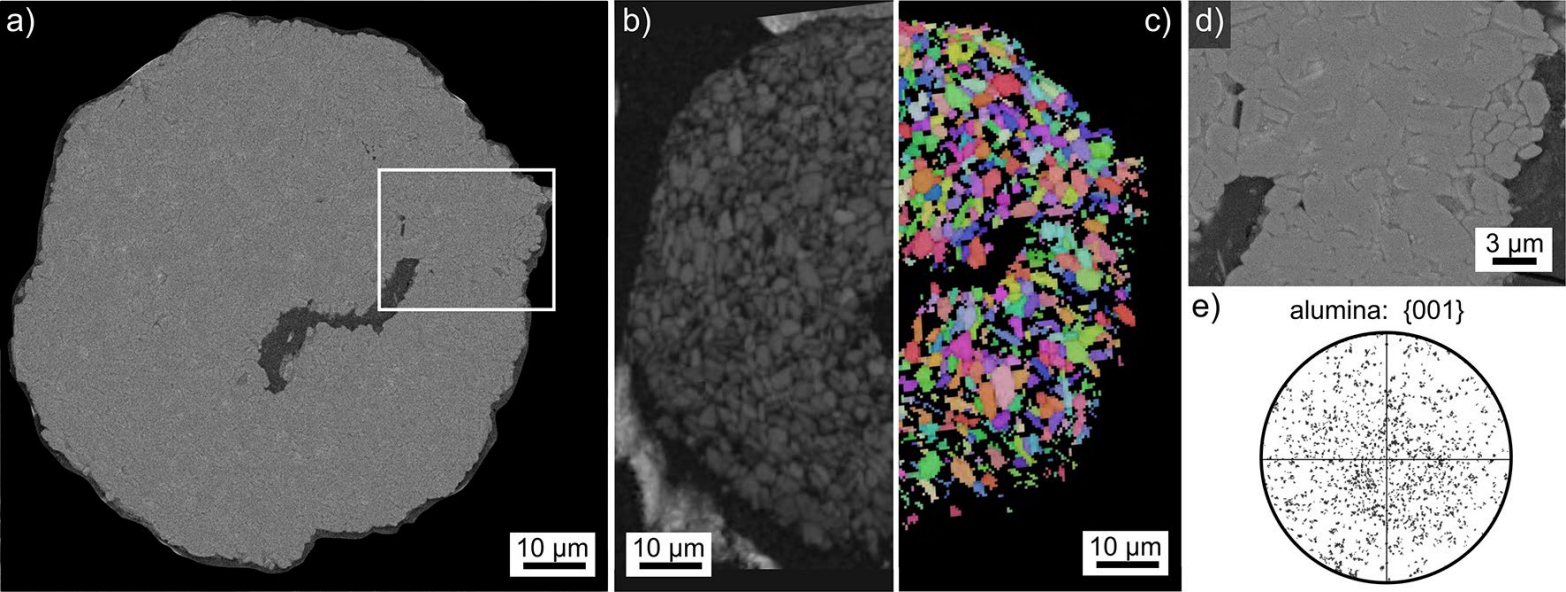
Results obtained from the crystalline body introduced in Fig. 2q: (**a**) SEM micrograph. (**b**) Partial BC map and (**c**) partial IPF + BC map of an EBSD-scan performed on the area. The IPF-legend for trigonal phases in Fig. 1 apply. (**d**) The frames area in greater detail and (**e**) the {001}-PF of α-alumina in the performed scan.

The final crystalline body introduced in Fig. 2q is only approximately spherical and results obtained from it are presented in Fig. 19. The outer boundary in the SEM-micrograph of Fig. 19a indicates that this structure was never melted during flame synthesis but only roughly sintered together in the flame. The partial BC map in Fig. 19b shows that EBSD-patterns could be obtained throughout the cross section, all of them were attributed to α-alumina. The partial BC + IPF-map of the other half presented in Fig. 19c shows that all grains show individual orientations. The framed area is presented in greater detail in Fig. 19d to visualize the microstructure while Fig. 19d presents the 001-PF of all data points attributed to α-alumina during the scan. As expected a texture is not indicated. This crystalline body is most likely the result of α-alumina agglomeration in the precursor powder and is included to show that such particles must be excluded if materials for e.g. optical applications are to be synthesized because it is unlikely that they will melt during flame synthesis or dissolve during subsequent sintering. Hence they would cause undesirable artifacts in the produced material.

In summary, a wide variety of production parameters is indicated to occur during flame synthesis by the presented results. In contrast to the conclusion that the crystals detected directly after flame synthesis result from incomplete melting instead of from melt crystallization due to an insufficient quenching rate^13,14^, the crystallization of YAG, YAP, YAM and probably Al_2_O_3_ observed in the microspheres detailed in the Figs. 3–7 and observed directly after flame synthesis clearly originate from crystallizing melts. Only the crystalline body in Fig. 19 probably results from incomplete melting. The conclusion of nm-sized crystals based on XRD-results^13^ also clearly does not apply to the presented microspheres.

It should, however, be noted that some of the presented microspheres are extremely rare. For example, a test scan covering several hundred microspheres was performed in order to locate the sphere presented in Fig. 2a which was the only crystallized sphere located during that measurement. The sphere featured in Fig. 2e is the only one found to contain YAM, and considering that this may have only formed due to the slight contamination with Si in a stria, its occurrence can be assumed to be extremely rare. It is likely that further types of crystallized microspheres exist as e.g. the type presented in Fig. 9e of Ref. ^18^ could not be located for EBSD-analysis during the current measurements.

Finally, a few statements made in previous publications should be commented: while polygon growth is relatively slow, dendritic growth occurs at high growth velocities when the growth front becomes instable. Describing the crystallized microspheres in Ref. ^17^ as cubic is incorrect: they certainly show a polygon morphology, but describing these as "cubic" can lead to an unfortunate mix of the polygon morphology and the cubic symmetry of the YAG crystal lattice. Standard SEM-micrographs cannot be used to judge the degree of microsphere crystallization^18,19^ as they do not contain any information whether a phase is crystalline or amorphous, as illustrated by the Figs. 8 and 9. Judging crystallite sizes based on such SEM-micrographs^19^ is also problematic as illustrated by the multiple dendritic and phase separated microstructures presented above: orientation domains may be larger than they appear if they are connected outside of the analyzed cross section.

Concerning the description of dendrites in some microspheres to be “needle-like”^20^, this analogy is misleading and only correct in specific cases where one growth direction is extremely preferred. The 3D-character of dendritic growth has e.g. been clearly illustrated by simulations^41^, the dendritic YAG-cube grown in a glass^10^ and the multiple cut planes through fresnoite dendrites featured in Ref. ^28^.

## Conclusions

The presented multitude of crystallized microspheres illustrates that flame synthesis is a process where strong deviations from the dominant chemical composition and thermal treatment can occur, albeit with very variable probabilities. Future publications concerning such microspheres should always discuss the amount in which the respective microspheres occur. All expected phases (YAG, YAP, YAM and α-alumina) were detected, although e.g. YAM was only found in a single microsphere containing an impurity of SiO_2_.

Polygon and dendritic crystal growth occur while spherical growth or viscous fingering where not detected. Multiple growth mechanisms of the same phase are sometimes observed indicating changing growth velocities, i.e. variable conditions during crystallization.

Due to the unique morphologies of the documented microspheres, they may subsequently be identified based on SEM-micrographs with a high probability. Including the widely available EDXS increases the probability for a correct identification if EBSD is not available. Furthermore, the crystallization models outlined for microspheres in the Y_2_O_3_–Al_2_O_3_-system are very likely transferable to the crystallization of glass microspheres in related systems.
